# GCDCA promotes hepatocellular carcinoma progression through S1PR2/PI3K/AKT-mediated polarization of M2-type macrophages

**DOI:** 10.3389/fimmu.2026.1640450

**Published:** 2026-02-23

**Authors:** Mengmeng Xue, Wei Yu, Kuizhi Zhang, Yu Chen, Luyao Zhang, Hengyan Zhang, Simin Lu, Min Tao, Haixin Yan, Lixin Wei, Gang Lv, Lu Gao, Li Zhang

**Affiliations:** 1 School of Pharmacy, Anhui Medical University, Hefei, China; 2 Department of Clinical Pharmacology, The Second Hospital of Anhui Medical University, Hefei, China; 3 Clinical Research Unit, Changhai Hospital, Naval Medical University, Shanghai, China; 4 Department of Urology, The Fourth Affiliated Hospital of Anhui Medical University, Hefei, China; 5 Department of Tumor Immunology and Gene Therapy Center, National Center for Liver Cancer, Naval Medical University, Shanghai, China; 6 Senior Department of General Surgery, The First Medical Center of Chinese People's Liberation Army (PLA) General Hospital, Beijing, China

**Keywords:** glycochenodeoxycholic acid, hepatocellular carcinoma, macrophage polarization, S1PR2 receptor, stemness

## Abstract

**Background:**

Disorders in bile acid metabolism are recognized as crucial mechanisms in the occurrence and development of hepatocellular carcinoma (HCC), a leading cause of cancer-related deaths worldwide. HCC progression is intricately linked to immune regulation within the tumor microenvironment (TME), particularly involving tumor-associated macrophages (TAMs) that modulate proliferation, invasion, and immune escape. Although glycochenodeoxycholic acid (GCDCA), a primary bile acid, is suspected to influence HCC, the specific mechanisms by which it affects the TME to drive cancer progression remain unclear.

**Methods:**

This study investigated the role of GCDCA in HCC progression using a combination of approaches. Single-cell sequencing was employed to analyze the TME and identify a highly malignant subpopulation of cancer stem cells (CSCs). In vivo experiments were conducted using a primary liver cancer model to assess the effect of GCDCA intervention on tumor progression and stemness. Mechanistic exploration focused on the role of the S1PR2 receptor, utilizing both macrophage and tumor cell systems to examine the S1PR2/PI3K/AKT signaling pathway and its influence on macrophage polarization.

**Results:**

Single-cell sequencing revealed a distinct subpopulation of CSCs with high malignancy within the TME. *In vivo*, GCDCA intervention significantly promoted liver cancer progression and enhanced the stemness of liver cancer cells. Mechanistically, GCDCA was found to activate the S1PR2 receptor on macrophages, triggering the S1PR2/PI3K/AKT signaling pathway. This activation induced macrophage polarization toward the M2 phenotype, which in turn promoted the growth and stemness of cancer stem cells.

**Conclusion:**

This study demonstrates that GCDCA drives HCC progression by inducing M2-type macrophage polarization via the S1PR2/PI3K/AKT signaling pathway, which subsequently enhances tumor cell stemness. These findings elucidate a novel mechanism by which bile acids remodel the TME to promote liver cancer, highlighting potential therapeutic targets within this pathway.

## 1 Introduction

Hepatocellular carcinoma (HCC) is the most common type of primary liver cancer and has become the fourth leading cause of cancer-related deaths worldwide (1). Liver cancer is often accompanied by chronic inflammation. In addition to inflammation caused by viral infections, long-term liver damage, human aging, and genetic mutations are causative factors for the development of liver cancer (2). Cholestatic liver disease caused by bile acid metabolism disorders has been recently intricately associated with the development of HCC. Abnormal accumulation of bile acids (BA) mediates hepatic inflammatory responses and induces hepatocellular damage, which can cause HCC (3). In addition, BA promote HCC progression by inducing stemness in tumor-initiating cells and promoting the generation of an immunosuppressive tumor microenvironment (4). Based on the intricate relationship between chronic inflammation and antitumor immune responses, investigating the mechanism of abnormally accumulated BA in the immune microenvironment of HCC may provide insights into the prediction of HCC biomarkers.

Cancer stem cells (CSCs) are a subpopulation of tumor cells possessing significant self-renewal capacity, differentiation potential, and pro-tumorigenic ability and exhibit a high degree of heterogeneity (5). CSC can provide drive tumorigenesis, cancer progression, recurrence, drug resistance, and metastasis (6). Different CSC subpopulations differ in their proliferation rates, drug resistance, and tumor-promoting properties (7). Early studies identified many CSC markers using cell sorting techniques. Among these, EPCAM, CD133, KRT19, CD24, and SOX9 are associated with aggressive HCC and poorer patient survival (8, 9). Therefore, reducing the stemness of HCC tissues may improve the prognosis of patients with HCC.

The maintenance of CSC stemness is dependent on the TME in which it exists. A complex TME exists in HCC tissues, and several components other than tumor cells are present in the TME, including immune cells (monocytes, neutrophils, dendritic cells, and natural killer cells), vascular endothelial cells, tumor-associated macrophages, fibroblasts, and liver-resident lymphocytes (B, CD8+ T, and CD4^+^ T cells) (10, 11). Increasing evidence suggests that complex interactions exist between tumor cells and other components of the TME and that tumor heterogeneity is intricately related to the TME (12). Abnormal energy depletion in the microenvironment and the accumulation of tumor cell metabolites affect the immune response of anti-tumor immune cells, creating an immunosuppressive TME and ultimately causing immune escape of tumor cells (13). Abnormal accumulation of BA induces the M2-type polarization of macrophages, generating an immunosuppressive TME conducive to tumor development (14). TAM are the primary immune cells with immunosuppressive effects in the TME, which are largely divided into M1-type macrophages that secrete pro-inflammatory factors, such as interleukin-6 (IL-6) and tumor necrosis factor-alpha (TNF-α), and M2 macrophages that secrete anti-inflammatory factors such as transforming growth factor-β (TGF-β) (15). Although advanced HCC cells can recruit TAM by secreting chemokines (16), recruited TAM can also interact with tumor cells to produce cytokines, such as IL6, that are beneficial to the maintenance of tumor cell stemness, and TAM recruitment has been implicated in the maintenance of tumor cell stemness (17). However, recent studies have demonstrated that the maintenance of CSC stemness depends more on M2 polarization than on TAM recruitment (18). M2 TAM are a major subpopulation of macrophages in the TME, which largely promote tumor invasion, metastasis, and immune escape, and several studies have focused on M2-type macrophages to inhibit their tumor-promoting effects (19). The loss of xanthine oxidoreductase in HCC-infected mice promotes macrophage M2 polarization and CD8^+^ T cell depletion, which consequently promotes HCC progression (20). M2 TAM promotes epithelial–mesenchymal transition and stemness by activating the Wnt/β-catenin signaling pathway in HCC cells by secreting chemokine ligand 17 (CCL17), which consequently promotes HCC cell proliferation and invasion (21). In addition, cytokines secreted by M2 TAM, such as TNF-α and TGF-β1, are intricately associated with tumor cell stemness (22, 23). Therefore, CSC modulation by targeting TAMs is an emerging option for tumor immunotherapy.

Abnormal metabolism is a significant feature of cancer and a potential target for therapy (14). The liver is a major metabolic organ and is the site of bile acid synthesis, and disorders of bile acid metabolism are intricately associated with the development of several diseases, such as HCC, colorectal cancer, inflammatory bowel disease, and type II diabetes mellitus (14, 24). The BA synthesized in hepatocytes are cholic acid (CA) and chenodeoxycholic acid (CDCA), which are two primary BA that can couple with taurine or glycine to form bile salts to enhance their amphiphilicity (3). Studies have demonstrated that high-fat diets cause elevated levels of bile salts in the intestine, and an increase in bile salts promotes the growth and proliferation of intestinal tumor cells, suggesting that excess bile salts may be a new type of carcinogen (25). Glycochenodeoxycholic acid (GCDCA), an important component of bile salts, is a hydrophobic and toxic bile salt with strong hepatocyte-damaging effects (26, 27). GCDCA is simultaneously the most abundant bile acid in human serum and is most significantly elevated in obstructive jaundice (28). GCDCA, an endogenous bile acid, is intricately associated with the development of HCC. Under cholestatic conditions, GCDCA may induce liver fibrosis by directly activating hepatic stellate cells (HSC) via the EGFR and MEK1/2 signaling pathways (29). In addition, GCDCA promotes HCC invasion and metastasis via the activation of AMPK/mTOR-dependent autophagy (30). Furthermore, GCDCA promotes chemoresistance in HCC by upregulating anti-apoptotic genes and downregulating pro-apoptotic genes (31). Although studies on GCDCA in HCC are ongoing, the specific mechanisms by which it promotes HCC progression need to be further explored. Here, we demonstrate that GCDCA promotes M2 macrophage polarization by activating the S1PR2/PI3K/AKT signaling pathway in macrophages, which facilitates the formation of an immunosuppressive TME and provides a new target for HCC therapy.

We revealed the critical function of GCDCA in HCC progression, particularly in inducing M2-type macrophage polarization through the activation of the S1PR2/PI3K/AKT signaling pathway. Our study suggests that GCDCA promotes the enhancement of tumor cell stemness by altering the TME, which consequently drives the malignant progression of HCC. These findings provide a new theoretical basis for immunotherapy of HCC, especially targeting the regulation of macrophages in the TME. The mechanism by which GCDCA is associated with HCC provides potential targets for the development of new therapeutic strategies, especially interventions in the immunosuppressive TME. Future studies will further explore the application of GCDCA and its related signaling pathways in the treatment of HCC to provide more effective therapeutic options for patients.

## 2 Materials and methods

### 2.1 Analysis of scRNA sequencing data

The single-cell RNA sequencing data generated in this study are available at the GeneExpression Omnibus (GEO, https://www.ncbi.nlm.nih.gov/geo/query/acc.cgi?acc=GSE218561), RNAsequencing data are available from the corresponding author on reasonable requestOther relevant data are within the manuscript and its Additional files. The “Seurat” R package was used for systematic analysis of this dataset. The analysis workflow primarily included the following steps: First, the CreateSeuratObject function was used to construct the Seurat object, with the initial filtering criteria set as follows: each gene must be expressed in at least three cells, and at least 200 genes must be detected in each cell subsequently, strict quality control was performed to retain high-quality cells with feature gene counts between 200 and 6,000 and mitochondrial gene proportions below 25%. Principal component analysis (PCA) was performed on the top 2,000 highly variable genes, and the top 10 principal components were selected for subsequent analysis using the ElbowPlot function. The FindClusters algorithm (with a resolution parameter of 0.5) was used for cell subpopulation identification, combined with t-distributed stochastic neighbor embedding (t-SNE) for dimensionality reduction and visualization. Finally, biological annotation of cell clusters was performed using known marker genes that successfully identified multiple cell types, including T cells, B cells, epithelial cells, mononuclear phagocytes, fibroblasts, endothelial cells, and NK cells.

### 2.2 InferCNV for single-cell analysis

InferCNV analysis was performed to detect large-scale somatic chromosomal copy number alterations (CNAs) using reference-normalized gene expression patterns (InferCNV; https://github.com/broadinstitute/InferCNV). We selected 1,000 endothelial cells, 1,000 fibroblasts, 2,000 mononuclear phagocytes, and 2,000 T/NK cells as reference controls to establish baseline expression. The inferCNV object was created using CreateInfercnvObject, and the analysis was conducted with the following default parameters: an expression cutoff of 0.1 (excluding lowly expressed genes), clustering by biological groups (cluster_by_groups = TRUE), denoising (denoise = TRUE), and without Hidden Markov Model smoothing (HMM = FALSE).

### 2.3 CytoTRACE score

We assessed the cellular differentiation state and stemness potential of malignant cells using the CytoTRACE algorithm, which predicts developmental potential based on gene expression signatures. We applied ScanoramaCT before analysis to mitigate batch effects across datasets. CytoTRACE scores were computed for all tumor cells using R, with values ranging from 0 (fully differentiated, low stemness) to 1 (least differentiated, high stemness), enabling the systematic characterization of the stemness hierarchy within the TME.

### 2.4 Gene set variation analysis

We performed Gene Set Variation Analysis using the GSVA package (v1.30.0) to evaluate differences in pathway activity among distinct cell types. Subsequently, the differential pathway activity across cell types was statistically assessed using the LIMMA package (v3.38.3), which employs an empirical Bayes approach to enhance the differential expression analysis.

### 2.5 Intercellular communication analysis

We used the Cellchat software package to infer interactions between different epithelial cell subpopulations and other cells in rat liver cancer tissues and predicted the strength of interactions between cells based on the ligands and receptors expressed by the cells.

### 2.6 Animal model

Male SD rats (6–8 weeks old, weighing 180–200 g) were purchased from Jihui Laboratory Animal Breeding Company, Ltd. (Shanghai, China). The AIN93M standard feed used in this study was purchased from Trophic Animal Feed High-tech Co., Ltd. (Nantong, China). The rats were kept in an animal facility free of any pathogens and fed DEN carcinogenic solution (0.95 g/mL diluted 100 ppm in triple-distilled water, MeilunBio^®^, China) daily for constructing a primary rat HCC model. 8 μmol/100 g GCDCA (Sigma-Aldrich, USA) was intraperitoneally injected twice a week starting from 12 weeks of DEN treatment, and 1.2% cholestyramine (MedChemExpress, USA) was fed to remove excess GCDCA *in vivo*. JTE-013 was injected intraperitoneally 2 h before GCDCA administration at a dose of 2 mg/kg body weight in the JTE-013 (TargetMol, China) interference model, starting at week 12 of DEN treatment. Each group consisted of 10 rats. All animal experiments were approved by the Animal Care Committee of the Naval Medical University. The ethics approval number was EDWLL-018.

### 2.7 Biochemical testing

Serum activities of alanine aminotransferase (ALT), aspartate aminotransferase (AST), and total bile acid (TBA) levels were analyzed using ALT, AST, and TBA assay kits (Rayto, China), respectively, according to the manufacturer’s instructions of the reagent vendors.

### 2.8 Hematoxylin and eosin staining

Sections were placed on a 60 °C roaster for 30 min and deparaffinized in water. Hematoxylin staining solution (Servicebio, China) was used for 8 min, and the sections were rinsed with water until they turned blue. After hydrochloric acid–alcohol differentiation, the slides were rinsed using deionized water, restained with eosin (Servicebio, China) for 2 s, rinsed with tap water, and dried. Finally, the slides were sealed with neutral gum and imaged under a microscope. Each experiment was conducted in triplicate.

### 2.9 Immunohistochemical staining and immunofluorescent staining

The slides were placed on a 60 °C roaster for 30 min, and sequentially dewaxed to water by xylene and gradient ethanol. An appropriate repair method was selected based on the type of antibody detected. The slides were washed thrice with PBS for 5 min each, and the tissues were labeled by drawing a circle with an immunohistochemical pen and closed for 30 min at room temperature using 3% H_2_O_2_ to block endogenous peroxidase. The slides were blocked with 3% BSA (Servicebio, China) for 30 min at 37 °C. After completion of primary antibody dilution with a universal antibody diluent (Epizyme, China), the primary antibody was added dropwise to the tissue block and incubated overnight at 4 °C in a wet box. The next day, the slides were removed from the refrigerator at 4 °C, rewarmed at room temperature for 10 min, the primary antibody was removed, and the slides were washed thrice with PBS for 5 min. The general secondary antibody was added dropwise to the tissue block, and the slides were incubated at 37 °C for 30 min. Slides were washed thrice with PBS after the incubation was completed, each time for 5 min. After completion of washing, the DAB color development solution (Genetech, China) was prepared for DAB development. We prepared trays containing tap water for the slides to terminate staining. The slides were re-stained with hematoxylin (Servicebio, China) for 6 min, rinsed under running water for several seconds, differentiated with hydrochloric acid–alcohol differentiation solution twice, each time for 1–2 s, and rinsed with running water until the blue color returned. The neutral resin was dropped onto the slide tissue block and sealed, allowing the slides to dry. Images were acquired using a microscope the next day for observation. The first day of immunofluorescence staining was the same as that of immunohistochemistry. A drop of the staining working solution was added to the tissue block on the second day, incubated at room temperature, protected from light for 10 min, washed with PBS, and the nuclei were stained blue with DAPI (Servicebio, China), incubated at room temperature, and protected from light for 10 min. Next, the tissue blocks were incubated with a tissue autofluorescence quencher (Servicebio) for 5 min at room temperature, protected from light, rinsed under running water, and sealed with drops of an anti-fluorescence quenching sealer (Servicebio). Images were captured using a single-photon confocal microscope (Stellaris 5; Leica, Germany). At least three random areas were selected for each slide to capture images. The following antibodies were used for immunohistochemistry: EPCAM (1:100, ab71916; Abcam), KRT19 (1:4000, 10712-1-AP; Proteintech), SOX9 (1:2000, ab185230; Abcam), CD163 (1:4000, ab213612; Abcam), S1PR2 (1:100, sc-365963; Santa Cruz Biotechnology), and CD68 (1:3000, ab31630; Abcam). The following antibodies were used for immunofluorescence: EPCAM (1:100, ab71916; Abcam), KRT19 (1:4000, 10712-1-AP; Proteintech), SOX9 (1:2000, ab185230; Abcam), CD163 (1:4000, ab213612; Abcam), CD206 (1:500, 18704-1-AP; Proteintech), and S1PR2(1:100, sc-365963; Santa Cruz Biotechnology). The quantitative analysis of immunohistochemical staining sections was performed using ImageJ 1.43. Each experimental group consisted of 3 slides, and 3 fields were randomly selected from each slide. Ultimately, the proportion of positive area for each experimental group was the average value of all the calculation results from the fields in that group.

### 2.10 Cell culture and treatment

The cells used in the experiments were purchased from the Cell Bank of the Chinese Academy of Sciences (Shanghai, China). The THP-1 cell line was prepared as RPMI 1640 complete medium using RPMI 1640 medium (BasalMedia, China) with 0.05 mM β-mercaptoethanol (Gibco, USA), 10% fetal bovine serum (FBS; Gibco, Invitrogen), and 1% penicillin and streptomycin (PS; YEASEN, China). F12K medium (Gibco, USA) was used for the NR8383 cell line, and DMEM (BasalMedia, China) was used for Huh7, RH35, and HTLA cell lines. Complete medium was prepared by adding 10% FBS and 1% PS to all cell lines, except the NR8383 cell culture medium, which was prepared by adding 15% FBS and 1% PS. All cells were incubated at 37 °C in an incubator containing 5% CO_2_.

The THP-1 cells were treated with 100 ng/mL PMA (MedChemExpress, USA) for 48 h. Fresh complete medium was replaced, and 20 ng/mL IL-4 (PeproTech, China) and IL-13 (TargetMol, China) were added to induce the polarization of THP-1 cells toward M2-type macrophages, and the cells were treated with GCDCA at a concentration of 25 μM for 24–48 h. The JTE-013 inhibitor (10 μM) was added 30 min before the addition of GCDCA. The NR8383 cells were treated with 25 μM GCDCA for 24–48 h and used for subsequent experiments. The JTE-013 inhibitor (10 μM) was added 30 min before GCDCA administration. The concentration of the inhibitor ketoconazole was 20 μM and was added 1 day before the addition of GCDCA. The inhibitor MeTC7 (5 μM) was added 1 day before the addition of GCDCA. Next, we co-cultured Huh7 cells with THP-1 cells, for which THP-1 cells were spread in the upper chamber using a 24 mm co-culture plate (Corning, USA) and treated with 100 ng/mL PMA for 48 h. Next, IL-4 and IL-13 (20 ng/mL) were added to induce macrophage polarization to M2.

macrophages, and 10 μM JTE-013 inhibitor and 25 μM GCDCA were administered according to the subgroups selected. After the polarization was completed, the cells were co-cultured with the human HCC cell line Huh7 in the lower chamber for 72 h. Next, we co-cultured RH-35 cells with NR8383 cells, for which NR8383 cells were spread in the upper chamber using a 24 mm co-culture plate (Corning, USA), and 10 μM JTE-013 inhibitor and 25 μM GCDCA were administered according to the grouping selection, and co-cultured with rat liver cancer cell line RH35 in the lower chamber for 72 h.

### 2.11 Cell counting kit-8

Each well was inoculated with 10^4^ cells in a 96-well plate (Corning, Corning, NY, USA), mixed using the cross method, and placed in an incubator at 37 °C with 5% CO_2_. After the cells attached to the wall, the old medium was aspirated. The cells were washed with PBS and treated with GCDCA for 24–48 h according to the concentration gradient set in the experiment. The supernatant was removed and replaced with 10% CCK8 solution (10 µL CCK8 + 90 µL medium; Servicebio, China), and incubated for 2 h at 37 °C in an incubator. The absorbance was measured at 450 nm using an enzyme marker (Zenyth1100). Cell viability was calculated using the following formula: cell viability (%) = (absorbance value of experimental group–absorbance value of blank group)/(absorbance value of control group–absorbance value of blank group) × 100%. Each experiment was conducted in triplicate.

### 2.12 Colony formation

Each well was inoculated with 500 cells in a six-well plate (Corning, USA) and placed in an incubator at 37 °C with 5% CO_2_. After the cells were attached to the wall on the second day, the cells were administered GCDCA and incubated at 37°C in an incubator containing 5% CO_2_ for 7 days, and the cell status was observed every day. After the cell colony grew to the appropriate size, the six-well plate was removed, the supernatant was discarded, the cells were washed twice with PBS, 1 mL of methanol (Macklin, China) was added to each well, and the plate was fixed for 15 min at room temperature. The methanol was removed, and the cells were stained with 0.5% crystal violet staining solution (Servicebio, China) for 30 min at room temperature. The six-well plate was gently rinsed with tap water to remove the excess crystal violet staining solution. The six-well plate was dried at room temperature, and images were acquired. The number of clones in each group was quantified using ImageJ 1.43. Each experiment was conducted in triplicate.

### 2.13 Cell sphere formation assay

The cell culture medium was prepared by adding 50 mL DMEM/F12 (Gibco, USA), 1 mL 1×B27 (Gibco, USA), 20 ng/mL EGF (MedChemExpress, USA), 10 ng/mL bFGF (TargetMol, China), and 0.4% BSA (Servicebio, China) to the medium. The medium was filtered with a 0.22 μm microporous filter membrane (Merck, Germany) and set aside. Each well was inoculated with 10^3^ cells in a low adsorption six-well plate (Corning). Next, 25 μM GCDCA was administered after the cells were stabilized on the next day. The cells were incubated in an incubator at 37 °C, 5% CO_2_ for 7 days, and observed daily, and imaged under a microscope after the cells became spherical. Each experiment was conducted in triplicate.

### 2.14 Western blotting

The lysate was prepared according to the ratio of PMSF protease inhibitor (100×, Servicebio, China): RIPA tissue cell rapid lysate (Servicebio) = 1:100; 200 μL of the protein lysate was added to each well of the six-well plate (Corning), and the plate was lysed on ice for 40 min. The cell lysate was collected and centrifuged for 15 min at 12,000 rpm at 4 °C. The supernatants were collected. The BCA working solution (Beyotime, China) was prepared, following which 25 µL of the supernatant was taken and mixed with 200 µL of the working solution (supernatant:working solution = 1:8), incubated at 37 °C for 30 min in the dark. Afterward, an enzyme meter (Zenyth1100) was used to measure the absorbance value of the samples at 595 nm, and subsequently, the concentration of proteins according to the standard curve. The supernatant 1/4 volume of 5× SDS-PAGE protein sampling buffer (Beyotime, China) was added, mixed well, and the protein was heated at 100 °C for 10 min to completely denature the protein. The precast gel (GenScript, China) was removed, the protein concentration was calculated, and electrophoresis was performed at 170 V for 45 min. A PVDF membrane (Merck, Germany) was activated in methanol and subsequently placed in a membrane equilibrium solution (GenScript) for 2 min. The gel was washed with deionized water, and the sponge, PVDF membrane, gel, and sponge were sequentially placed in a transfer folder to transfer the membrane. After transfer, the membrane was placed in a protein-free rapid sealing solution (Servicebio) for 15 min at room temperature, and the primary antibody was incubated overnight. The next day, the membrane was removed, the strips were washed with 1×TBST (Servicebio), and the corresponding secondary antibody (Servicebio) was incubated with the primary antibody source. After incubation for 90 min at room temperature, the strips were washed again, and the chemiluminescent horseradish peroxidase substrate (Merck, USA) was mixed in a 1:1 ratio, immersed in the mixture for a few seconds, and developed using a Tanon automated chemiluminescent image analyzer system (Tanon-4600). Each experiment was repeated thrice. The following antibodies were used in the experiments: GAPDH (BioWord, AP0063, 1:5000), EPCAM (1:1000, ab71916, Abcam), KRT19 (1:3000, 10712-1-AP, Proteintech), SOX9 (1:1000, ab185230, Abcam), CD163 (1:1000, ab213612, Abcam), ARG1 (1:5000, 16001-1-AP, Proteintech), CD206 (1:500, 18704-1-AP, Proteintech), AKT(1:1000, 4685S, CST), p-AKT(1:2000, 66444-1-Ig, Proteintech), PI3K(1:5000, 60225-1-Ig, Proteintech), and p-PI3K (1:5000, AF3242, Affinity).

### 2.15 Real-time PCR

Total RNA was extracted from the cells using a HiPure Total RNA Plus Micro Kit (MGBio, China) according to the manufacturer’s instructions of the reagent company. The cDNA was synthesized using Hifair^®^ V One-Step RT-gDNA Digestion SuperMix for qPCR Kit (YEASEN, China). The total reaction system was 20 µL, including 15 µL of RNase-free H₂O and total RNA, 4 µL 5× Hifair^®^ One-Step RT SuperMix (YEASEN, China), and 1 µL gDNA Remover Mix (YEASEN, China). The cDNAs obtained from this synthesis were used for subsequent PCR experiments. Real-time fluorescence quantitative PCR was performed using the Hieff UNICON^®^ Universal Blue qPCR SYBR Green Master Mix (YEASEN, China) as described by the manufacturer’s instructions. The total reaction system was 20 µL, including 8 µL of RNase-free H_2_O, 1 µL of cDNA, 0.5 µL of forward primer, 0.5 µL of reverse primer, and 10 µL of the SYBR Green Master Mix (YEASEN, China). The reaction procedure was standardized: 2 min of pre-denaturation at 95 °C (1 cycle), followed by denaturation at 95 °C for 10 s (40 cycles), annealing and extension at 60 °C for 30 s (40 cycles), and finally melting curve analysis (1 cycle). Information on the primers used in the experiments is provided in the Supplementary Material. Gene expression was quantified using the 2^-ΔΔCt^ method. The gene expression values were normalized to those of GAPDH. Each experiment was repeated thrice.

### 2.16 Enzyme-linked immunosorbent assay

THP-1 and NR8383 cells were seeded in six-well plates at a density of 1 × 10^6^ cells/well. THP-1 cells were treated with PMA and pre-treated with GCDCA for 24–48 h after adhering to the plate. Subsequently, the cell culture supernatants were collected, and the concentrations of IL-6 (Thermo Fisher Scientific, USA), TNF-α (Thermo Fisher Scientific), IL-10 (Thermo Fisher Scientific), TGF-β (Cloud-Clone Corp, China), and VEGF (Multi Science, China) in the supernatants were determined according to the manufacturer’s instructions. Each sample was subjected to three repetitions of the experiment.

### 2.17 Flow cytometry

THP-1 cells were collected during the logarithmic growth phase by centrifugation at 1000 rpm for 5 min. The cell precipitate was collected and washed twice with precooled PBS. The cells were dissolved in PBS containing an Fc receptor blocker (BD, USA), incubated at 4 °C for 15 min to block non-specific binding sites. The cells were dissolved in 100 µL of pre-cooled PBS, and fluorescently labeled CD163 (Biolegend, USA) and CD206 antibodies (Biolegend, USA) were added, incubated at 4 °C in the dark for 30 min, and subsequently washed thrice with pre-cooled PBS. After the incubation, the cells were suspended in 100 µL of Cyto-Fast™ Fix/Perm cell fixation and membrane disruption solution (Biolegend) at room temperature for 20 min. Next, 1 mL of 1× Cyto-Fast™ Perm washing solution (Biolegend) was added and the above antibody incubation steps were repeated to detect anti-CD163 and CD206 antibodies in the cells. The cells were resuspended in PBS in a flow cytometry tube and detected using a flow cytometer (LSRFortessa, BD, USA). Data analysis was conducted using the FlowJo 10.8.1 software. After data collection, median fluorescence intensity (MFI) of CD206 and CD163 was calculated for quantitative analysis. All experiments were performed in three independent replicate samples.

### 2.18 Tango GPCR detection system

An appropriate number of HTLA cells was inoculated into 96-well cell culture plates. The next day, when the cell density reached about 80%, 200 ng of the GPCR-Tango plasmid solution (dissolved in 200 μL of DMEM) was added, mixed with 400 ng of polyethyleneimine in an equal volume of DMEM, and incubated at room temperature for 20 min. After incubation, the transfection mixture was added to HTLA cells for transfection. After 24 h of transfection, the culture medium was replaced. The agonist ADRB1 (positive control) for GCDCA and S1PR2 receptor was added according to the experimental group settings. Next, 50 μL of the Bright-Glo solution (Promega, USA) was added to each well. Next, the wells were incubated at room temperature for 20 min, and the luminescence intensity was measured using a microplate luminometer (LB963, Centro). The multiplicity of activation for each sample was calculated as the relative luminescence units (RLU) for each condition divided by the control RLU for the medium only.

### 2.19 General transcriptome sequencing

GCDCA-treated and untreated NR8383 cells were collected for transcriptome sequencing, which was performed by LC-Bio Technologies Co., Ltd. (Hangzhou, China). Each group consisted of three replicates. For data analysis, genes that were not detected in more than 25% of the samples were first removed, and the raw count values were normalized. Differentially expressed genes were calculated using edgeR 3.38.4 with the screening criteria of FDR < 0.05 and |log2FC| > log2(1.5). Finally, pathway enrichment analysis was performed using clusterProfiler 4.4.4.

### 2.20 Statistical analysis

All data are expressed as mean ± standard deviation (SD). Data were analyzed using R version 4.2.1 and GraphPad Prism 8.0. Differences between two and multiple groups of data were compared by Student’s *t*-test and one-way analysis of variance (ANOVA), respectively. Statistical significance was set at *P* < 0.05, implying that the results were statistically significant. All experiments were repeated thrice.

## 3 Results

### 3.1 Single-cell RNA sequencing reveals the presence of malignant tumor stem cells in liver cancer tissues

**Figure 1:**
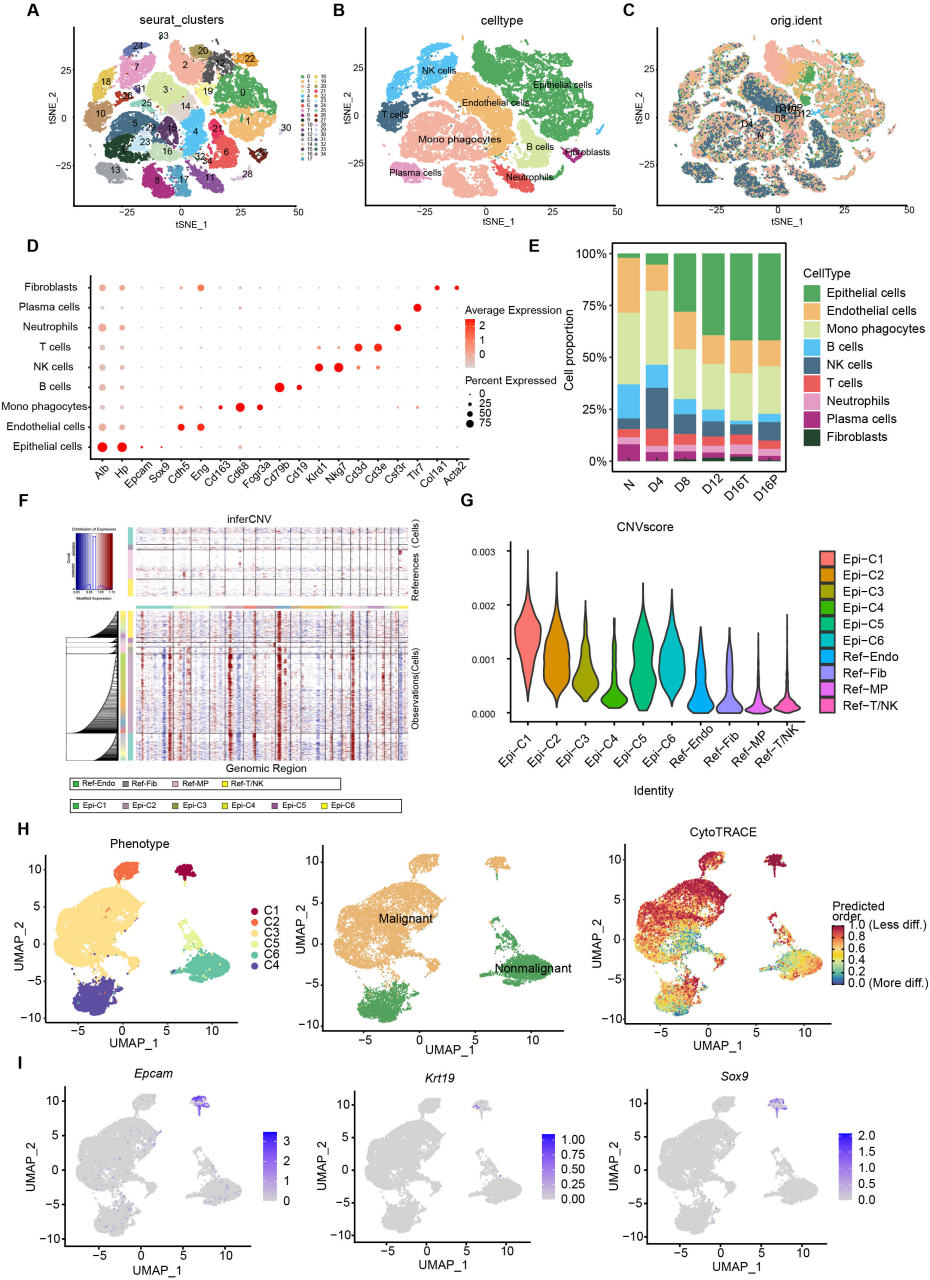
Single-cell RNA-seq reveals the presence of malignant cancer stem cells in liver cancer tissues. **(A)** T-distributed stochastic neighborhood embedding (tSNE) plot showing clustering results of individual cells in the liver of DEN-treated rats. **(B)** tSNE plot showing cell types in the liver of DEN-treated rats (epithelial cells, endothelial cells, B cells, monophagocytes, NK cells, T cells, neutrophils, plasma cells, and fibroblasts). **(C)** tSNE plots showing individual cells analyzed, colored according to the time point of DEN treatment. **(D)** Dot plots showing the expression of marker genes in the indicated cell types. Dot size indicates the percentage of cells expressing the marker gene in each cell type, and dot color indicates the average expression of the marker gene in each cell type. Red indicates high expression of the marker gene, whereas gray indicates low expression. **(E)** Percentage of each cell type in rat liver tissues at different time points of DEN-induced carcinoma. **(F)** Stratified heat map showing large-scale copy number variations in epithelial cell subpopulations versus spiked control cells (endothelial, fibroblastic, mononuclear phagocytic, NK, and T cells). **(G)** Violin plots were used to score the degree of variability of epithelial cell subpopulations in rat liver tissues. **(H)** CytoTRACE score of epithelial cell subpopulations. **(I)** Expression of stemness markers *Epcam*, *Krt19*, *Sox9* in epithelial cell subpopulations. (N, control; D4, DEN-induced carcinoma 4W; D8, DEN-induced carcinoma 8W; D12, DEN-induced carcinoma 12W; D16P, DEN-induced carcinoma 16W paracancerous tissues; D16T, DEN-induced carcinoma 16W tumor tissues; C, epithelial cell subpopulations).

We analyzed the composition of the TME and the degree of malignancy of tumor cells in a DEN-induced rat model of primary HCC using single-cell RNA sequencing to assess the degree of malignancy of tumor tissues and explore the potential mechanisms promoting the development of HCC. We performed single-cell RNA sequencing of rat liver tissues at different time points (0, 4, 8, 12, and 16 weeks) to assess DEN-induced carcinoma. Single-cell clustering caused the clustering of all liver cells into 35 cell clusters (**Figure 1A**). Several major cell types in the liver were identified after clustering analysis based on classical markers: epithelial cells, endothelial cells, B cells, mononuclear phagocytes, NK cells, T cells, neutrophils, plasma cells, and fibroblasts, as well as the distribution of subpopulations of different cell types at different time points (**Figures 1B, C, D**). We conducted a proportion analysis of different cells to reveal the expression characteristics and distribution of different cell types in the liver tissues of DEN-induced carcinogenic rats. The relative abundance of each cell type in the liver cancer microenvironment was observed, which also reflected the dynamic changes in the composition of cells during the occurrence of liver cancer (**Figure 1E**). This suggests that the dynamic remodeling of the immune microenvironment is accompanied by tumorigenesis and development. Next, we used inferCNV to conduct further clustering analysis of epithelial cells. InferCNV analysis is a common method used to assess cellular malignancies. The degree of variation in the chromosomal copy number of target cells was determined by comparing the chromosomes of target cells with those of control cells. A darker color resulted in more CNV variation and higher cell malignancy. Epithelial cells were clustered and analyzed to obtain six cell subpopulations, and inferCNV analysis was performed using endothelial cells, fibroblasts, and T cells as reference cells. The C1 and C2 subpopulations had more significant variations. We calculated the CNV score and compared it with the reference cells, and found that the epithelial C1 and C2 subpopulations were significantly different from the reference cells; therefore, these cell subpopulations were defined as malignant cells (**Figures 1F, G**). HCC CSCs were identified using the CytoTRACE score to predict the differentiation status of the epithelial cells. CytoTRACE can count genes at the single-cell level to predict the differentiation status and direction of cells. A higher CytoTRACE score leads to higher stemness of the cells and a lower degree of differentiation (32). The CytoTRACE scores were highly heterogeneous in the tumor tissues, with the highest scores in the C1 and C2 subgroups (**Figure 1H**). The expression of *Epcam*, *Sox9*, and *Krt19*, stemness markers, was analyzed in the subpopulations of epithelial cells; the C1 subpopulation was found to highly express *Epcam*, *Sox9*, and *Krt19* (**Figure 1I**). The above experimental results demonstrated that a subpopulation of CSC had high malignancy in rat HCC tissues and that the CSC subpopulation highly expressed *Epcam*, *Sox9*, and *Krt19*.

### 3.2 GCDCA promotes HCC progression and enhances hepatocellular carcinoma cell stemness

**Figure 2:**
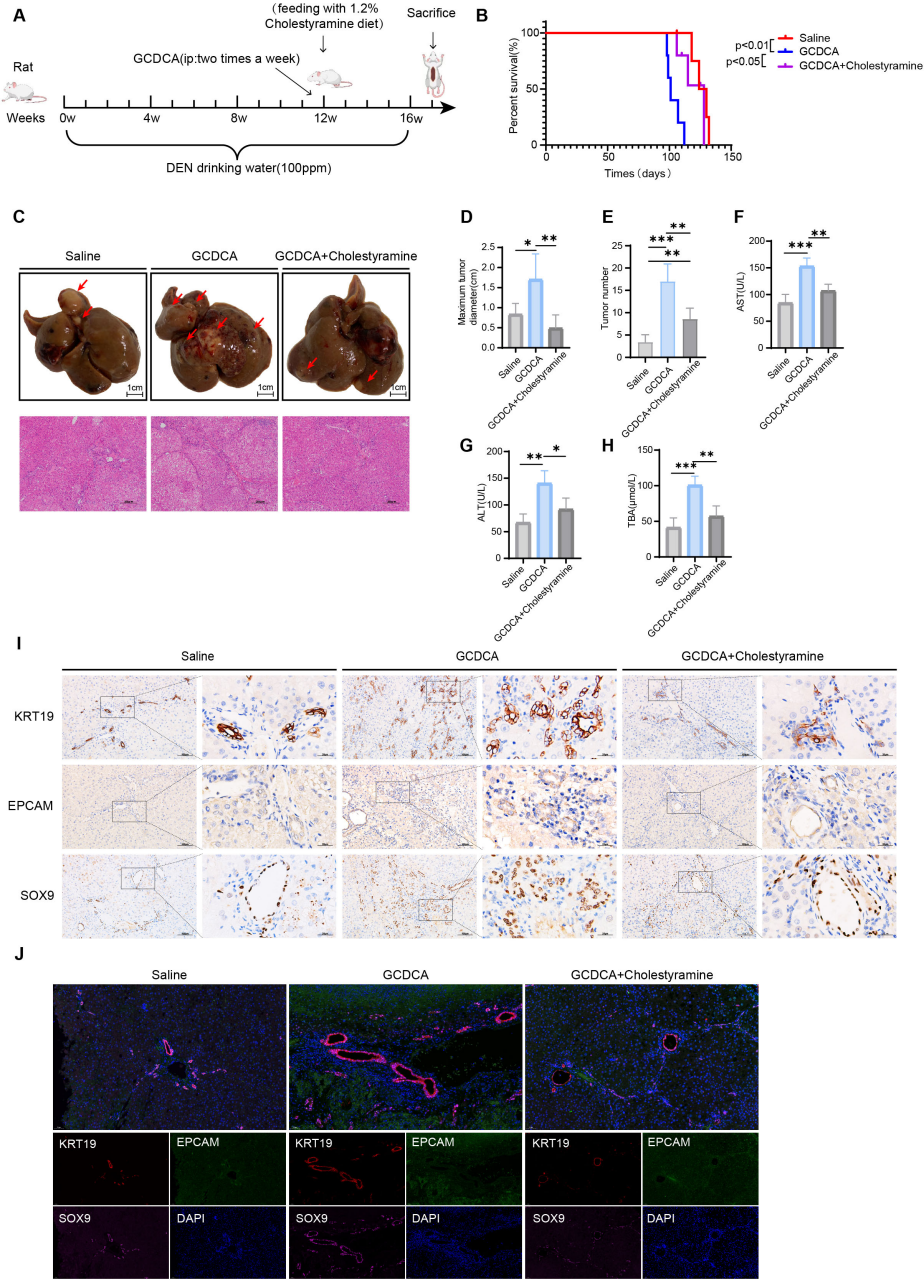
GCDCA promotes the HCC progression and increases hepatocellular carcinoma cell stemness. **(A)** Flow chart of animal experiments. **(B)** Kaplan–Meier plots of DEN-induced primary hepatocellular carcinoma in rats after administration of GCDCA and cholestyramine (*n* = 5 in each group). **(C)** Representative images of liver tumors in different groups of rats (scale bar = 1 cm) and HE-stained images (scale bar = 200 μm). **(D, E)** Maximum diameters of liver tumors in rats in different groups and the number of tumors for quantitative analysis (*n* = 5 in each group). **(F, H)** Serum AST and ALT activities and TBA levels in different groups of rats (*n* = 4 in each group). **(I)** Representative immunohistochemical staining of KRT19-, EPCAM-, and SOX9-positive cells in the livers of different groups of rats, scale bar = 100 μm (*n* = 3 in each group). **(J)** Representative immunofluorescence staining of co-localized positive cells of KRT19 (red), EPCAM (green), and SOX9 (pink) in liver CSCs of different groups of rats. Cell nuclei were stained with DAPI (blue). Scale bar = 50 μm. *p < 0.05, **p < 0.01, and ***p < 0.001, n.s., not significant.

Several patients with HCC develop jaundice in the late stages, indicating that cholestasis is a very common pathological symptom in these patients. Patients with HCC complicated by jaundice have a significantly shorter survival period and poor prognosis (33, 34). GCDCA is a toxic bile acid synthesized by the human liver. When the liver is damaged, disturbances in bile acid metabolism cause an abnormal accumulation of bile acids, further aggravating liver injury. We used a rat primary HCC GCDCA intervention model to verify whether the toxic bile acid GCDCA has a tumor growth-promoting effect *in vivo*. The rat model was intraperitoneally injected with 8 μmol/100 g of GCDCA at week 12 of DEN-induced carcinogenesis to humanize the rat bile acid pool. Injections were administered for 4 consecutive weeks to mimic the effect on liver cancer in the presence of cholestasis. The other group was simultaneously fed diets enriched with 1.2% cholestyramine (an anion-exchange resin that binds to intestinal BA and hinders their reabsorption) at week 12, and the effects of GCDCA on DEN-induced primary HCC in rats were observed after 16 weeks (**Figure 2A**). Rats administered intraperitoneal injections of GCDCA had significantly shorter survival compared to those treated with cholestyramine (**Figure 2B**). This indicated that GCDCA promotes the progression of liver cancer. Oncological index tests were also conducted on the liver tissues of each rat group. The maximum diameter and number of liver tumors in the GCDCA-treated rats were significantly higher than those in the control group (**Figures 2C, D, E**). HE staining revealed severe liver damage in this group of rats (**Figure 2C**). These rats also exhibited higher activities of Aspartate Aminotransferase (AST) and Alanine Aminotransferase (ALT), and higher levels of Total Bile Acid (TBA), resulting in more severe liver injury (**Figures 2F, G, H**). To further assess the malignancy of the tumor tissue, immunohistochemistry and immunofluorescence staining were performed for the stemness markers EPCAM, SOX9, and KRT19. The positive signal was stronger in the liver after GCDCA treatment (**Figures 2I, J**; **Supplementary Figure 1A**). Thus, toxic GCDCA promotes HCC progression and enhances liver cancer tissue stemness.

### 3.3 GCDCA does not directly promote stemness in hepatocellular carcinoma cells

**Figure 3:**
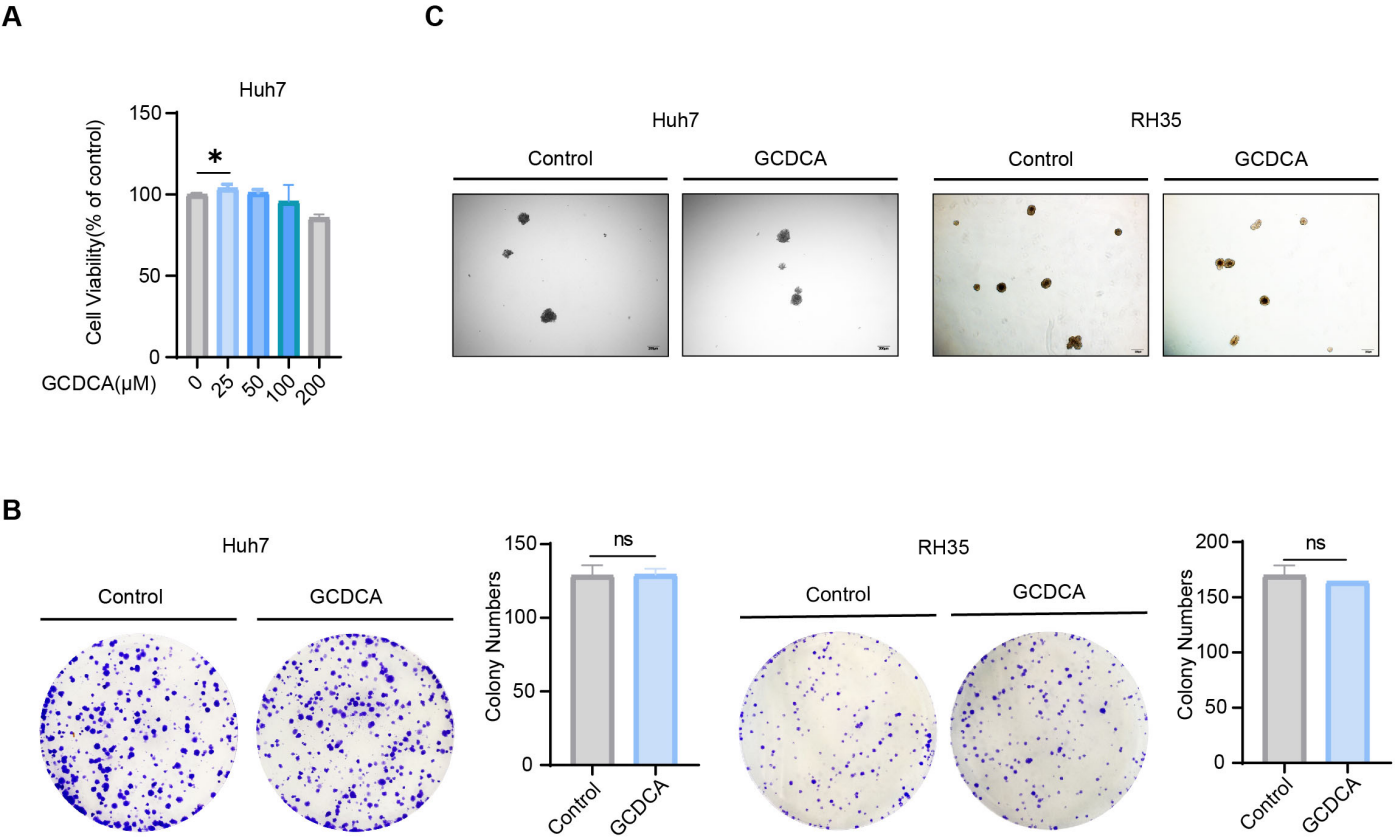
GCDCA does not directly promote stemness in hepatocellular carcinoma cells. **(A)** Relative viability values of hepatocellular carcinoma cells in different concentrations of GCDCA (*n* = 3 in each group). **(B)** Effect of GCDCA on clonogenic ability of different hepatocellular carcinoma cell lines. **(C)** Effect of GCDCA on sphere-forming ability of different hepatocellular carcinoma cell lines. Scale bar = 50 µm. Cell clones were counted using ImageJ 1.43. *p < 0.05, **p < 0.01, and ***p < 0.001, n.s., not significant.

We directly treated the human HCC cell line Huh7 and the rat HCC cell line RH35 with GCDCA to investigate the mechanism by which GCDCA promotes the development of HCC. The results of the cell proliferation assay demonstrated that GCDCA promoted HCC cell proliferation (**Figure 3A**). The cloning and sphere-forming assays revealed that GCDCA had no direct effect on HCC cell stemness (**Figures 3B, C**). The effect of GCDCA in promoting stemness in HCC cells may be realized indirectly by acting on other components of the TME, rather than through direct action on HCC cells.

### 3.4 Single-cell RNA sequencing reveals malignant stem cell-M2 macrophage interactions in the tumor microenvironment

**Figure 4:**
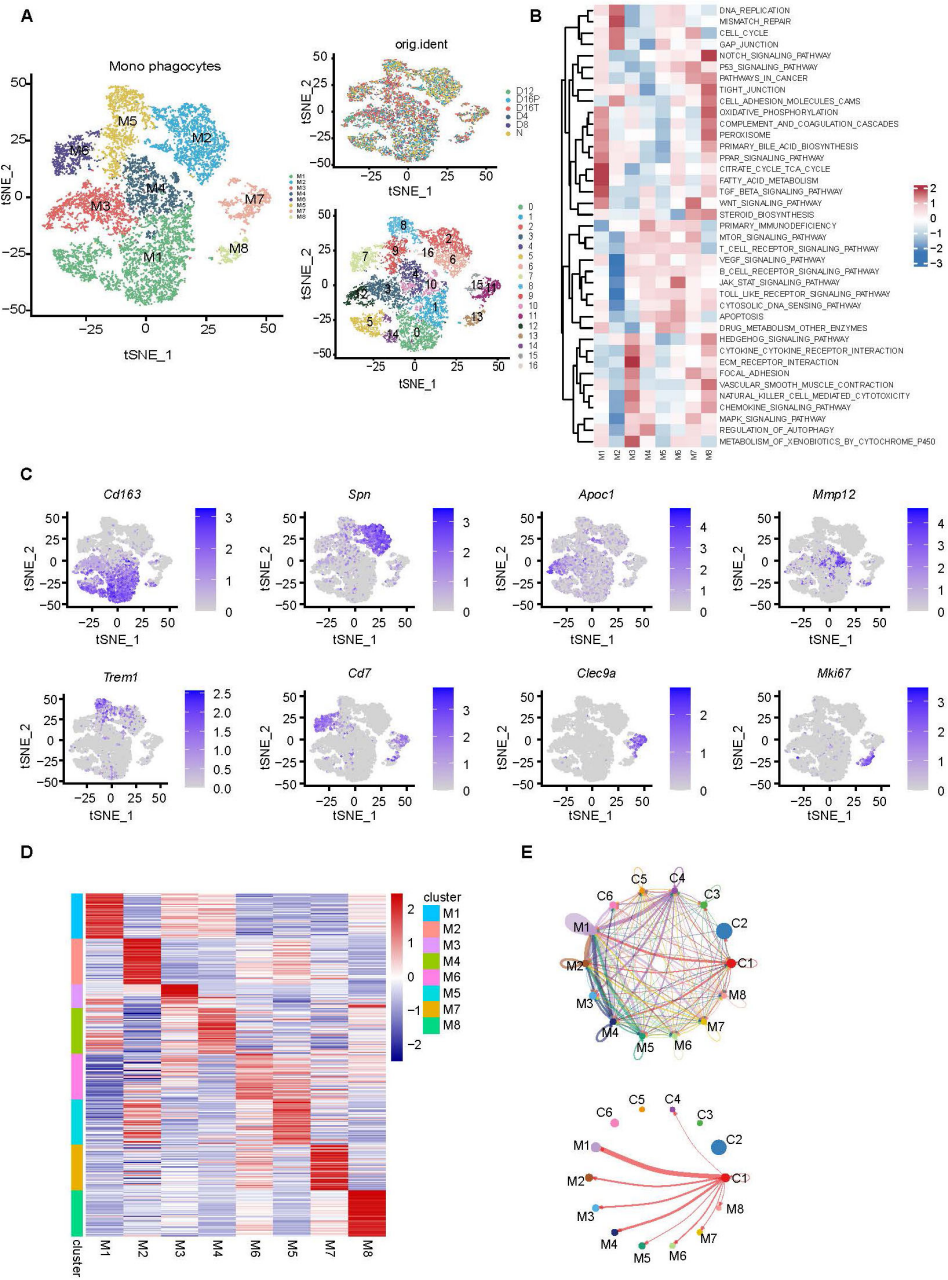
Single-cell RNA-seq reveals malignant stem cell-M2 macrophage interactions in the tumor microenvironment. **(A)** tSNE plot showing the clustering results of mononuclear phagocytes in rat liver. **(B)** GSVA enrichment analysis of pathways associated with different macrophage subpopulations. **(C)** tSNE plot showing the distribution of *Cd163*, *Spn*, *Apoc1*, *Mmp12*, *Trem1*, *Cd7*, *Clec9a*, and *Mki67* gene expression in macrophage subpopulations. **(D)** Heat map showing differentially expressed genes between eight macrophage subpopulations. **(E)** Network diagram of epithelial cell subpopulations interacting with different macrophage subpopulations. Circle colors represent different cell types, and circle sizes represent cell numbers; the larger the circle, the greater the number of cells. Cells emitting arrows express ligands, cells pointing to them express receptors, and the thickness of the line indicates the number of ligand–receptor pairs in both cells. M, macrophage subset; C, epithelial cell subset.

Recently, an increasing number of studies have demonstrated that macrophage diversity is implicated in the occurrence and development of tumors. Macrophages exhibit distinct functions at different tumor stages. During the initial stages, they promote epithelial cell carcinogenic mutations by generating an inflammatory microenvironment and activating immune responses. They undergo phenotypic polarization in advanced stages, transforming from pro-inflammatory to tissue repair-like phenotypes, and regulate malignant processes such as tumor invasion, migration, angiogenesis, and immunosuppression (35). To identify macrophage subpopulations that interact with the C1 subpopulation in the CSC described above, all mononuclear phagocytes in the liver were further classified into 16 cell clusters, which yielded eight major macrophage subpopulations, and all types of macrophages were present in the livers at different stages of DEN-induced cancer (**Figure 4A**). We conducted gene set variation analysis (GSVA) to further analyze the functional characteristics of each macrophage subpopulation. This method converts the expression of a single gene into an enrichment score for a gene set, thereby reflecting the differential activity of specific functional pathways or biological processes. GSVA revealed that the macrophage subpopulation associated with bile acid biosynthesis and immunosuppressive signaling pathways was the M1 population (**Figure 4B**). The tSNE plot demonstrating the expression of Cd163, Spn, Apoc1, Mmp12, Trem1, Cd7, Clec9a, and Mki67 in the macrophage subpopulations revealed that the M1 subpopulation of macrophages highly expressed Cd163 in M2 macrophages (**Figure 4C**). Strong interactions between C1 subpopulations and M2-type macrophages in CSC were revealed using heat maps (**Figure 4D**) and by conducting a cellular interaction analysis (**Figure 4E**). These results indicated a strong interplay between CSC and M2 macrophages in the TME of HCC. M2 macrophages in the TME can interact with CSCs to promote stemness, proliferation, and migration of tumor cells by secreting cytokines, microRNAs, and chemokines (36). This was demonstrated by our findings. Therefore, GCDCA may promote M2 polarization of macrophages by acting on macrophages, which consequently enhances the stemness of tumor cells.

### 3.5 GCDCA induces M2-type macrophage polarization and thus promotes tumor cell stemness

**Figure 5:**
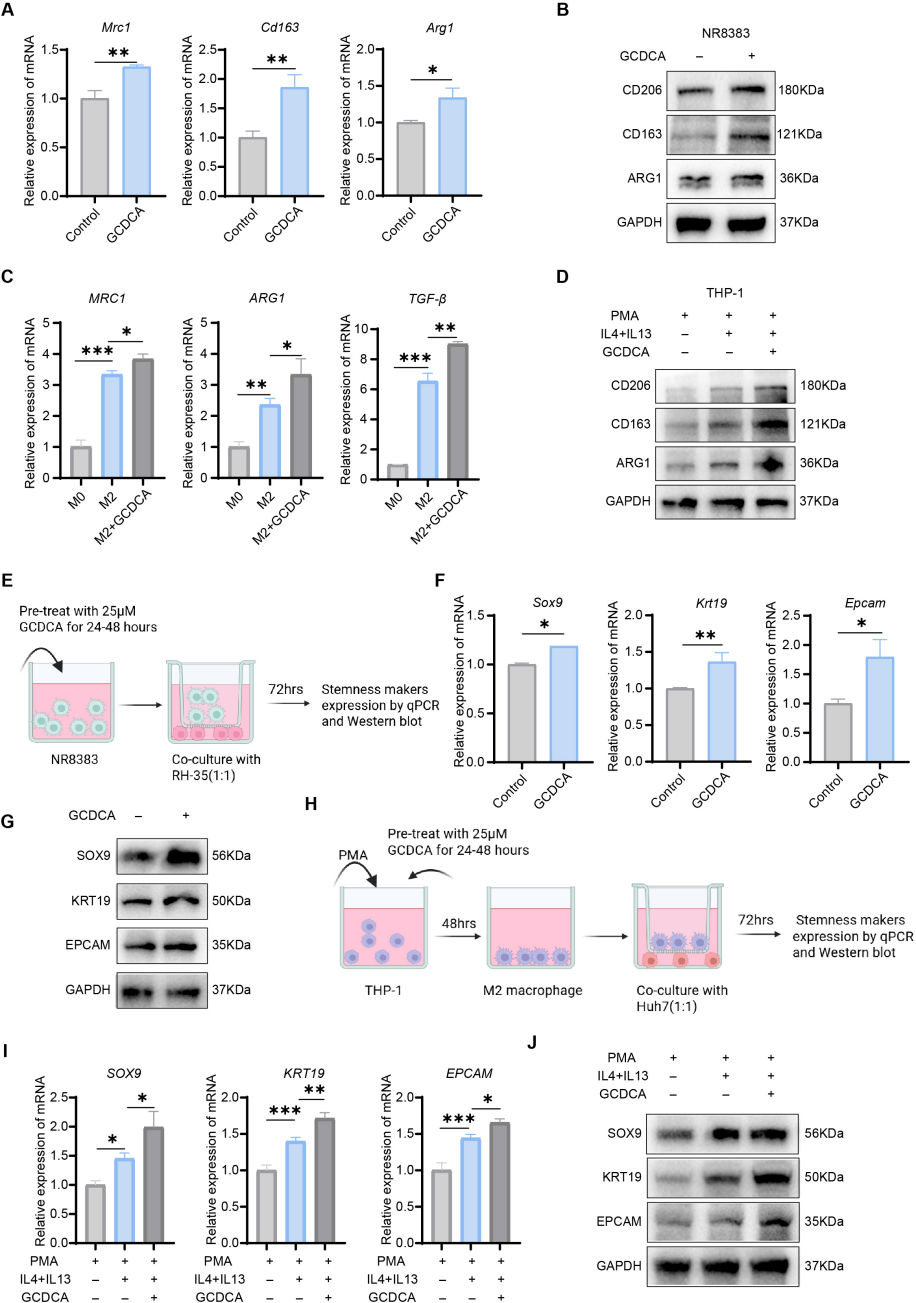
GCDCA-induced polarization of M2 macrophages promotes stemness in hepatocellular carcinoma cells. **(A)** Expression of M2 macrophage marker genes *Mrc1*, *Cd163*, *Arg1* in NR8383 cells after GCDCA treatment (n = 3 in each group). **(B)** Expression of CD206, CD163, ARG1 proteins in NR8383 cells. **(C)** Expression of M2 macrophage marker genes *MRC1*, *ARG1*, and *TGF-β* in THP-1 cells after GCDCA treatment (n = 3 in each group). **(D)** Expression of CD206, CD163, ARG1 proteins in THP-1 cells. **(E)** Co-culture pattern of NR8383 and RH35 cells. **(F)** Expression of stemness genes *Krt19*, *Epcam*, and *Sox9* in RH35 cells after co-culture with GCDCA-treated NR8383 cells (n =3 in each group). **(G)** Expression of KRT19, EPCAM, and SOX9 proteins in RH35 cells. **(H)** Co-culture pattern of THP-1 and Huh7. **(I)** Expression of stemness genes *KRT19*, *EPCAM*, and *SOX9* in Huh7 cells after co-culture with GCDCA-treated THP-1 cells (n = 3 in each group). **(J)** Expression of KRT19, EPCAM, and SOX9 proteins in Huh7 cells. GAPDH was used as an internal reference. *p < 0.05, **p < 0.01, and ***p < 0.001, n.s., not significant.

We first treated rat macrophage NR8383 with GCDCA to investigate whether GCDCA can induce M2 polarization in macrophages, and found that the mRNA expression of the M2 macrophage marker genes *Mrc1*, *Cd163*, and *Arg1* was upregulated (**Figure 5A**). GCDCA induced the polarization of rat macrophages, as confirmed by the increased expression of CD206, CD163, and ARG1 proteins (**Figure 5B**). This conclusion was validated in the human acute monocytic leukemia cell line THP-1. THP-1 was first treated with PMA for 48 h to induce differentiation of THP-1 into macrophages, which were subsequently induced to form M2-type macrophages with IL-4 and IL-13, and continued to induce M2-type macrophage polarization in the presence or absence of GCDCA treatment, and we determined the macrophage phenotype using qRT-PCR. The mRNA expression of M2 macrophage marker genes *MRC1*, *ARG1*, and *TGF-β* was significantly up-regulated in GCDCA-treated macrophages compared to controls (**Figure 5C**). Western blotting revealed the expression of CD206, CD163, and ARG1 proteins consistent (**Figure 5D**). Flow cytometry revealed an elevated proportion of M2-type macrophages in the treatment group (**Supplementary Figures 2A, B**). These results indicated that GCDCA promotes macrophage polarization to the M2 phenotype. To further confirm this conclusion, we conducted an ELISA experiment to detect the expression of cytokines IL-6, TNF-α, IL-10, TGF-β, and VEGF in the supernatant of cells after GCDCA treatment. We found that IL-6 and TNF-α, pro-inflammatory factors, were downregulated (**Supplementary Figures 2C, D**), whereas IL-10, TGF-β, and VEGF, immunosuppressive cytokines, were upregulated (**Supplementary Figures 2C, D**). These results suggest that GCDCA promotes macrophage polarization to the M2 type, consequently generating a tumor-immunosuppressive microenvironment and facilitating tumor immune escape.

Several molecules, including EPCAM, KRT19, SOX9, and CD133, have been used as CSC biomarkers (37–40). Genetic features associated with *EPCAM* can be used as key predictors of survival in HCC (41). An *in vitro* co-culture system of tumor cells and macrophages was constructed to investigate whether the GCDCA-induced polarization of M2 macrophages could promote tumor cell stemness. NR8383 cells were cultured in the upper chamber of six-well Transwell plates, and after completion of cell-induced differentiation, the cells were co-cultured with rat tumor cells RH35 in the lower chamber for 72 h (**Figure 5E**). Macrophages in the GCDCA-treated group significantly enhanced the expression of HCC cell stemness genes *Epcam*, *Sox9*, and *Krt19*, and upregulated the corresponding protein levels compared with the control group (**Figures 5F, G**). We investigated whether GCDCA exerted the same effect on human cells. We cultured THP-1 cells in the upper chamber of transwell six-well plates, induced THP-1 differentiation using PMA for 48 h, and added IL-4 and IL-13 to induce M2-type macrophage polarization, and finally provided GCDCA intervention for 24–48 h. M2-type macrophages that had completed the induced differentiation were co-cultured for 72 h with the human HCC cell line Huh7 (**Figure 5H**). Next, the expression of the stemness marker genes *EPCAM*, *KRT19*, and *SOX9* was detected by qRT-PCR in HCC cells, and the expression of stemness marker genes was significantly upregulated, similar to that in NR8383 cells. The same was performed to validate the protein levels, and the western blot results were consistent with the qRT-PCR results (**Figures 5I, J**). The results of the above experiments demonstrated that GCDCA-treated macrophages strongly induced stemness in HCC cells.

### 3.6 GCDCA induces M2-type polarization in macrophages through activation of S1PR2 receptor

**Figure 6:**
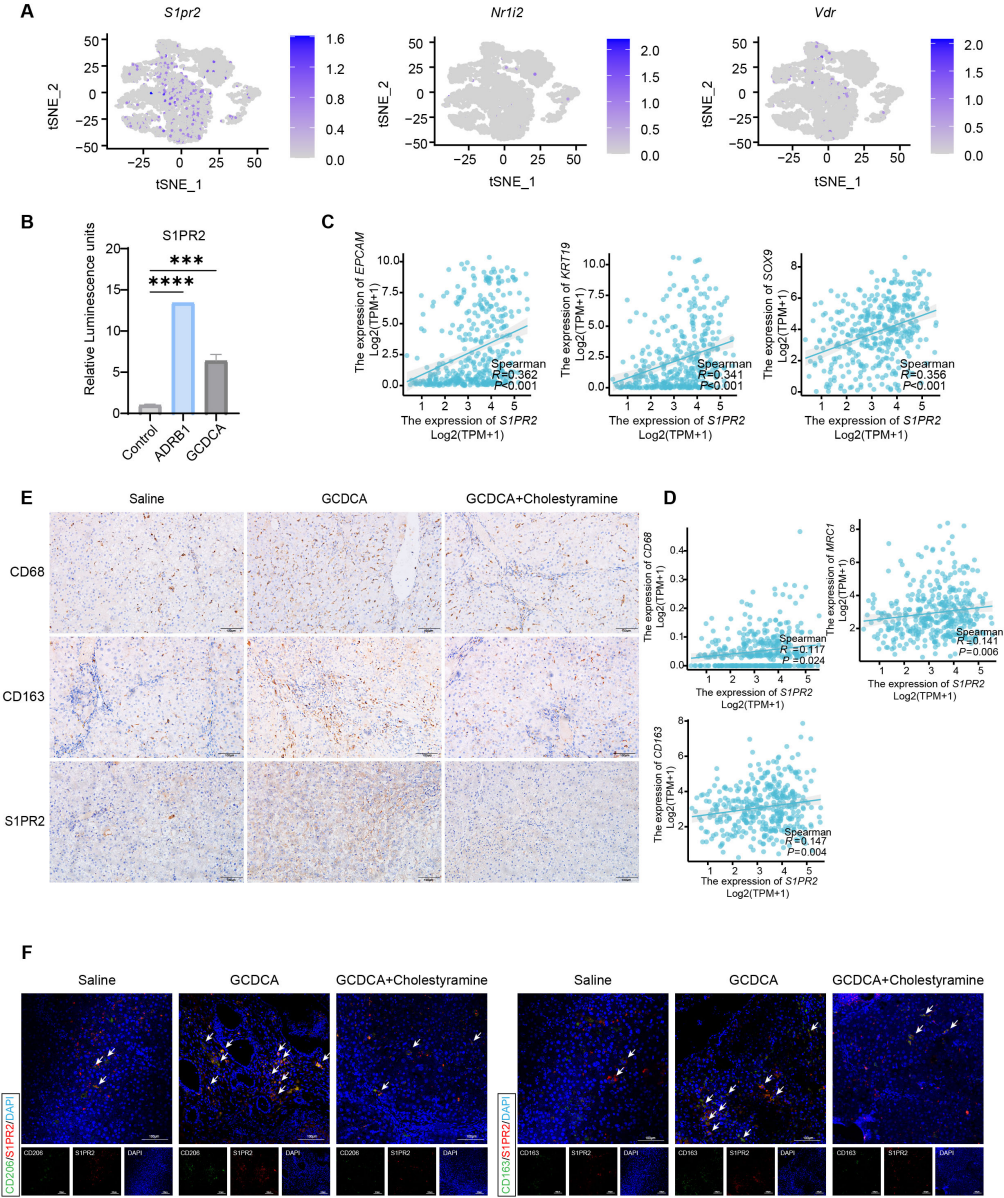
GCDCA induces M2-type polarization in macrophages through activation of S1PR2 receptors. **(A)** Single-cell sequencing to analyze bile acid receptor expression in M2 macrophages. **(B)** Activation of the S1PR2 receptor by GCDCA was detected using the Tango GPCR assay (n = 3 in each group). **(C, D)** TCGA database analysis of *S1PR2* correlation with *KRT19*, *EPCAM*, *SOX9*, *CD68*, *MRC1*, *CD163*. **(E)** Representative immunohistochemical staining of CD68, CD163, and S1PR2-positive cells in the livers of different groups of rats, scale bar = 100 μm (n = 3 in each group). **(F)** Representative immunofluorescence staining of S1PR2 (red) co-localized with CD163- (green) and CD206-(green) positive cells in the livers of different groups of rats, scale bar = 100 μm. *p < 0.05, **p < 0.01, and ***p < 0.001, n.s., not significant.

Bile acid is an important signaling molecule that maintains homeostasis and energy metabolism by activating bile acid receptors (42). Two membrane receptors exist for common bile acids, the G protein-coupled receptor (TGR5) and sphingosine-1-phosphate receptor 2 (S1PR2), and four nuclear receptors, the farnesol X receptor (FXR), pregnane X receptor (PXR), constitutive androstane receptor (CAR), and vitamin D3 receptor (VDR) (42, 43). Previous studies have demonstrated that GCDCA activates macrophage polarization, enhancing tumor cell stemness. However, exactly which bile acid receptor in macrophages is activated is not yet known. The expression of bile acid receptors in macrophage subpopulations was analyzed using single-cell sequencing to further investigate which receptor GCDCA activates. Only three bile acid receptors, S1PR2, VDR, and NR1I2, were expressed in rat macrophages, with the S1PR2 receptor being widely expressed in different macrophage subpopulations, whereas the other two bile acid receptors were less expressed (**Figure 6A**). To verify our hypothesis, we used the Tango GPCR assay to test whether GCDCA activates S1PR2, and found that GCDCA activated the S1PR2 receptor (**Figure 6B**). An analysis of the TCGA database revealed that S1PR2 expression was positively correlated with the expression of both M2 macrophage markers and stemness genes in human HCC tissues (**Figures 6C, D**). CD68, a TAM marker, is strongly associated with HCC staging (17). Immunohistochemical staining revealed that the expression of CD68, CD163, and S1PR2 was higher in GCDCA-treated liver tissues, whereas the expression of CD68, CD163, and S1PR2 was down-regulated in cholestyramine-treated liver tissues (**Figure 6E**; **Supplementary Figure 3A**). These results further confirmed that GCDCA accelerates HCC progression. Immunofluorescence co-localization of the S1PR2 receptor with the M2-type macrophage markers CD206 and CD163 demonstrated a significant increase in the number of S1PR2-positive M2-type macrophages after GCDCA treatment compared to controls, a phenomenon that was reversed by the addition of cholestyramine (**Figure 6F**). Thus, S1PR2 mediates M2-type macrophage polarization and enhances tumor cell stemness.

### 3.7 Pharmacological inhibition of S1PR2 by JTE-013 reverses GCDCA-induced stemness

**Figure 7:**
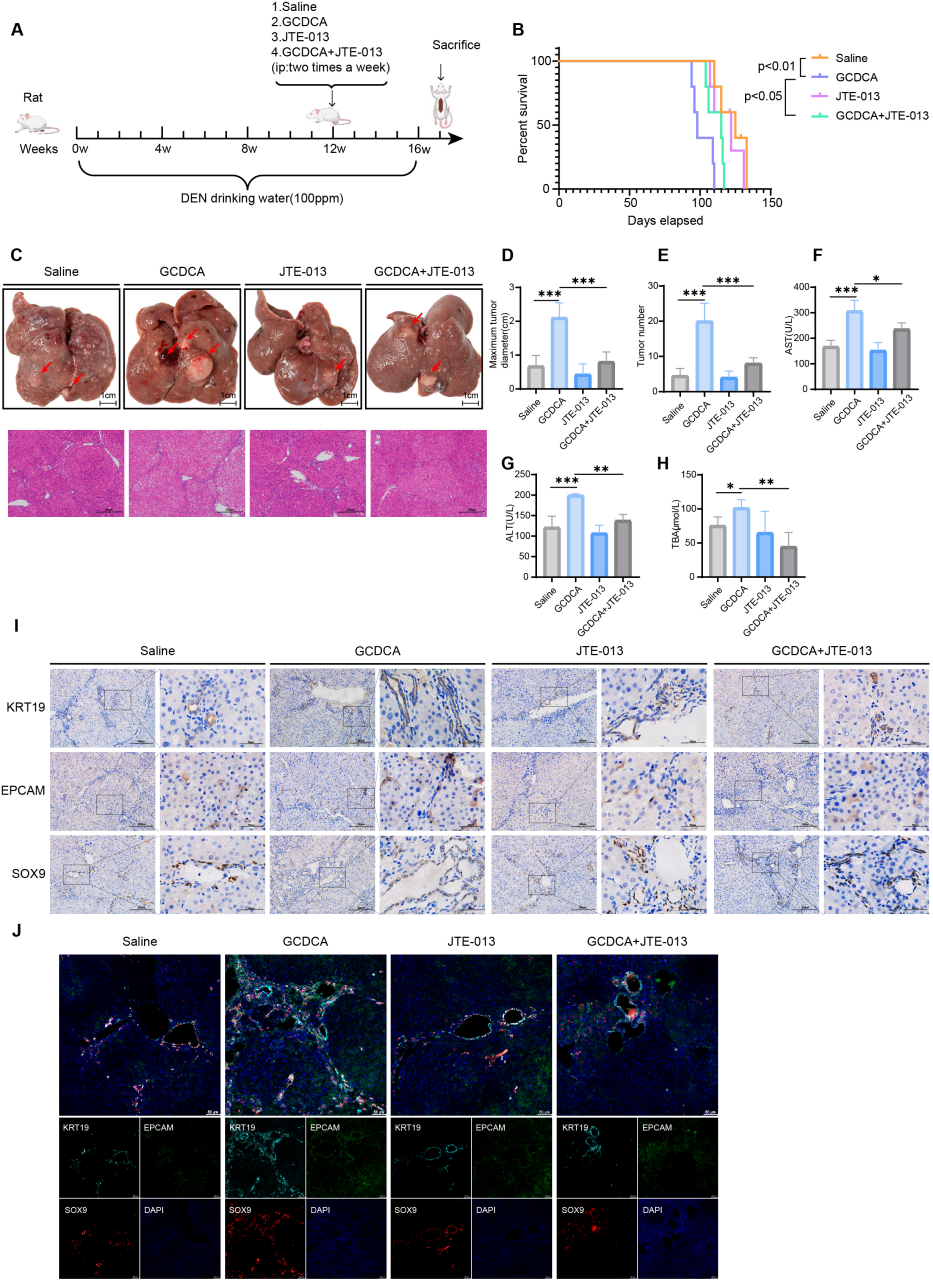
Pharmacological inhibition of S1PR2 by JTE-013 reverses GCDCA-induced stemness. **(A)** Flow chart of animal experiments. **(B)** Kaplan–Meier plots of DEN-induced primary hepatocellular carcinoma in rats after administration of GCDCA and the receptor inhibitor JTE-013 (*n* = 5 in each group). **(C)** Representative images of liver tumors in different groups of rats (scale bar = 1 cm) and HE-stained images (scale bar = 200 μm). **(D, E)** Analysis of maximum diameter and number of tumors (n = 4 in each group). **(F–H)** Serum AST and ALT activities and TBA levels in different groups of rats (n = 4 in each group). **(I)** Representative immunohistochemical staining of KRT19, EPCAM, and SOX9-positive cells in the livers of different groups of rats, scale bar = 200 μm (n = 3 in each group). **(J)** Representative immunofluorescence staining of KRT19, EPCAM, and SOX9 co-localized positive cells in the livers of different groups of rats, scale bar = 50 μm. *p < 0.05, **p < 0.01, and ***p < 0.001, n.s., not significant.

To further confirm the conclusion that GCDCA promotes M2 polarization of macrophages through activation of S1PR2 receptors, and thus enhances the stemness of HCC cells, we further verified this *in vivo* by establishing a rat primary HCC GCDCA intervention model, whereby GCDCA was injected intraperitoneally in the 12th week of carcinogenesis. The other group was pretreated with JTE-013 for 2 h and then administered GCDCA intervention to block the S1PR2 receptor *in vivo* (**Figure 7A**). Samples were collected after 16 weeks of DEN-induced cancer, and the results showed that the survival of rats pretreated with JTE-013 was prolonged compared to that of the group treated with GCDCA alone (**Figure 7B**). The maximum diameter and number of rat liver tumors were significantly reduced after pretreatment with JTE-013 compared to those in the group treated with GCDCA alone, whereas no effect was observed in the group treated with JTE-013 alone (**Figures 7C, D, E**). HE staining results demonstrated that liver injury was alleviated in rats after JTE-013 pretreatment (**Figure 7C**). In addition, rats in the JTE-013 pretreated group exhibited lower activities of ALT and AST, and TBA levels than those in the GCDCA-treated group (**Figures 7F, G, H**). Immunohistochemistry and immunofluorescence staining for EPCAM, SOX9, and KRT19, which are stemness markers, revealed that the positive signals in the liver were attenuated after JTE-013 pretreatment (**Figures 7I, J**; **Supplementary Figure 4A**). These results confirmed that pharmacological inhibition of S1PR2 by JTE-013 reversed GCDCA-induced stemness enhancement in tumor cells. To confirm that the action specificity of S1PR2 antagonist JTE-013 stems from its regulation of macrophages, we treated hepatocellular carcinoma cells with the same concentration of JTE-013 alone. Using CCK-8 and clonogenic assays, we investigated the effects of JTE-013 on the proliferation and survival capacity of hepatocellular carcinoma cells. Results indicate that JTE-013 itself does not directly inhibit the proliferation and survival of hepatocellular carcinoma cells (**Supplementary Figures 4B, C**).

### 3.8 Inhibition of S1PR2 receptor reverses GCDCA-induced polarization of M2-type macrophages and tumor cell stemness enhancement

**Figure 8:**
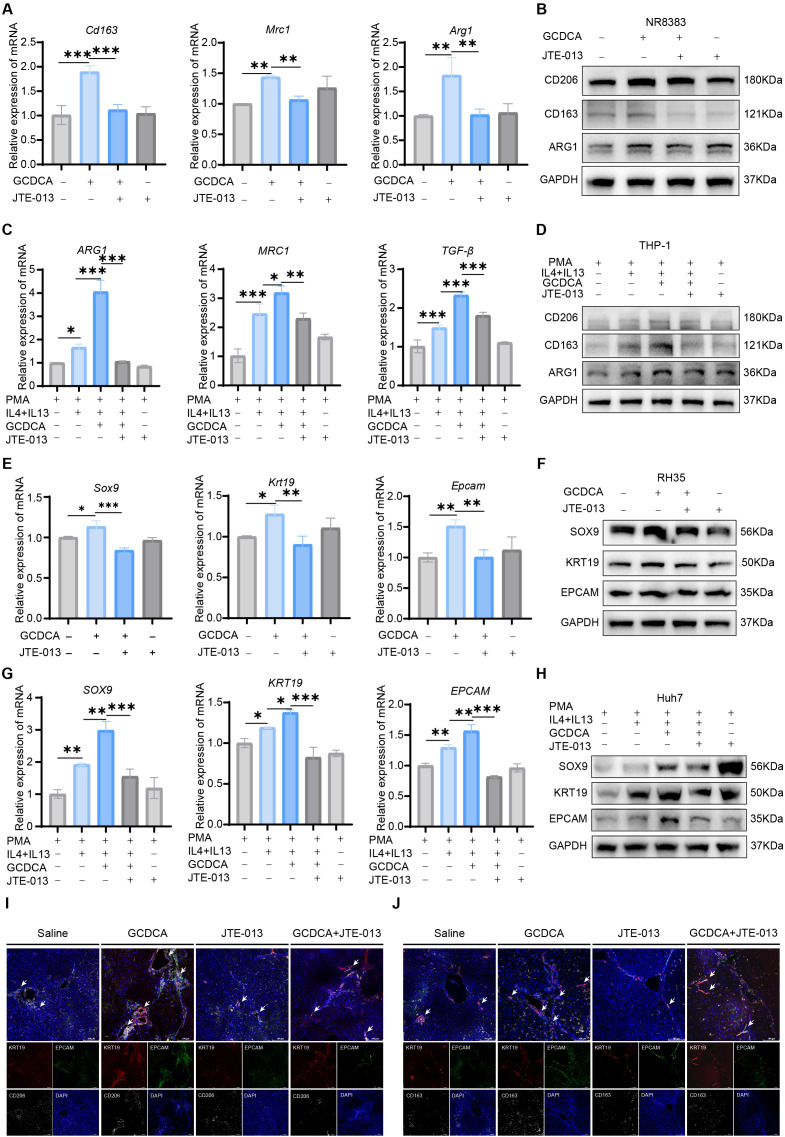
Inhibition of S1PR2 receptor reverses GCDCA-induced polarization of M2 macrophages. **(A)** Expression of M2 macrophage marker genes *Mrc1*, *Cd163*, and *Arg1* in NR8383 cells after inhibition of the S1PR2 receptor (n = 3 in each group). **(B)** Expression of CD206, CD163, and ARG1 proteins in NR8383 cells after inhibition of the S1PR2 receptor. **(C)** Expression of M2 macrophage marker genes *MRC1*, *ARG1*, and *TGF-β* in THP-1 cells after inhibition of the S1PR2 receptor (n = 3 in each group). **(D)** Expression of CD206, CD163, and ARG1 proteins in THP-1 cells after inhibition of the S1PR2 receptor. **(E)** Expression of stemness genes *Krt19*, *Epcam*, and *Sox9* in RH35 cells after co-culture with GCDCA-induced NR8383 cells pretreated with JTE-013 (n = 3 in each group). **(F)** Expression of KRT19, EPCAM, and SOX9 proteins in RH35 cells after co-culture with GCDCA-induced NR8383 cells pretreated with JTE-013. **(G)** Expression of stemness genes *KRT19*, *EPCAM*, and *SOX9* in Huh7 cells after co-culture with GCDCA-induced THP-1 cells pretreated with JTE-013 (n = 3 in each group). **(H)** Expression of KRT19, EPCAM, and SOX9 proteins in Huh7 cells after co-culture with GCDCA-induced THP-1 cells pretreated with JTE-013. **(I-J)** Representative immunofluorescence staining of M2 macrophage markers CD206 (white) and CD163 (white) co-localized with KRT19- (red) and EPCAM- (green) positive cells in the livers of different groups of rats, scale bar = 100 μm. GAPDH is used as an internal reference. *p < 0.05, **p < 0.01, and ***p < 0.001, n.s., not significant.

Previous studies have demonstrated that GCDCA induces the polarization of M2 macrophages by activating S1PR2 receptors; therefore, *in vitro* inhibition of S1PR2 receptors has the opposite effect. To further verify our hypothesis, the NR8383 cell line was pretreated with the inhibitor JTE-013 for 30 min to block S1PR2 receptors, and subsequently, cells were treated with GCDCA for 24–48 h. The expression of the M2-type macrophage marker genes *Mrc1*, *Cd163*, and *Arg1* was downregulated in the JTE-013-pretreated group compared to the GCDCA-treated group alone, whereas no significant difference was noted in the JTE-013-treated group compared to the control group (**Figure 8A**). This was also confirmed by the reduced expression of the corresponding proteins (**Figure 8B**). Similarly, this finding was confirmed on human macrophages, where the expression of M2 macrophage marker genes *MRC1*, *ARG1*, and *TGF-β* was significantly down-regulated in the JTE-013-pretreated group compared to the GCDCA alone group (**Figure 8C**). The expression of CD206, CD163, and ARG1 proteins was consistent with their (**Figure 8D**). Previous studies have demonstrated that S1PR2 is highly expressed in macrophages, whereas that of the other two receptors, PXR and VDR, is relatively low. We treated NR8383 and THP-1 cells with their corresponding receptor inhibitors, namely, ketoconazole and MeTC7, to eliminate the influence of these two receptors (PXR and VDR) on the polarization of M2-type macrophages. These inhibitors did not have a significant inhibitory effect on macrophage polarization (**Supplementary Figures 5A–H**). These results suggest that GCDCA promotes the polarization of M2-type macrophages by activating the S1PR2 receptor.

NR8383 cells pretreated with JTE-013 with or without GCDCA treatment were co-cultured *in vitro* with the rat HCC cell line RH35 to investigate the effect of S1PR2 receptor inhibition on the stemness of hepatocellular carcinoma cells. Compared with the GCDCA-treated group alone, the expression of the HCC cell stemness genes *Epcam*, *Sox9*, *Krt19*, and their corresponding proteins was significantly downregulated in the JTE-013 pretreated group (**Figures 8E, F**). The same was again confirmed in human macrophages, where THP-1 cells were induced to differentiate, and PMA, IL-4, and IL-13 inducers were added to induce M2-type macrophage polarization. JTE-013 was administered 30 min before GCDCA treatment, and the treated macrophages were co-cultured with the human HCC cell line Huh7. These results were similar to those described above, and inhibition of the S1PR2 receptor suppressed the polarization of M2-type macrophages and further inhibited the expression of stemness marker genes (**Figures 8G, H**). Immunofluorescence staining of the M2-type macrophage markers CD206 and CD163 and the stemness markers EPCAM and KRT19 demonstrated a significant increase in the number of CSCs and peripheral aggregation of M2-type macrophages in GCDCA-treated liver tissue. After treatment with JTE-013, the number of CSCs and M2-type macrophages decreased (**Figures 8I, J**). These results further confirm that GCDCA promotes the polarization of M2-type macrophages by activating the S1PR2 receptor on macrophages and that M2-type macrophages interact with tumor cells, consequently enhancing the stemness of tumor cells.

### 3.9 GCDCA induces macrophage polarization toward M2 via the S1PR2/PI3K/AKT signaling pathway

**Figure 9:**
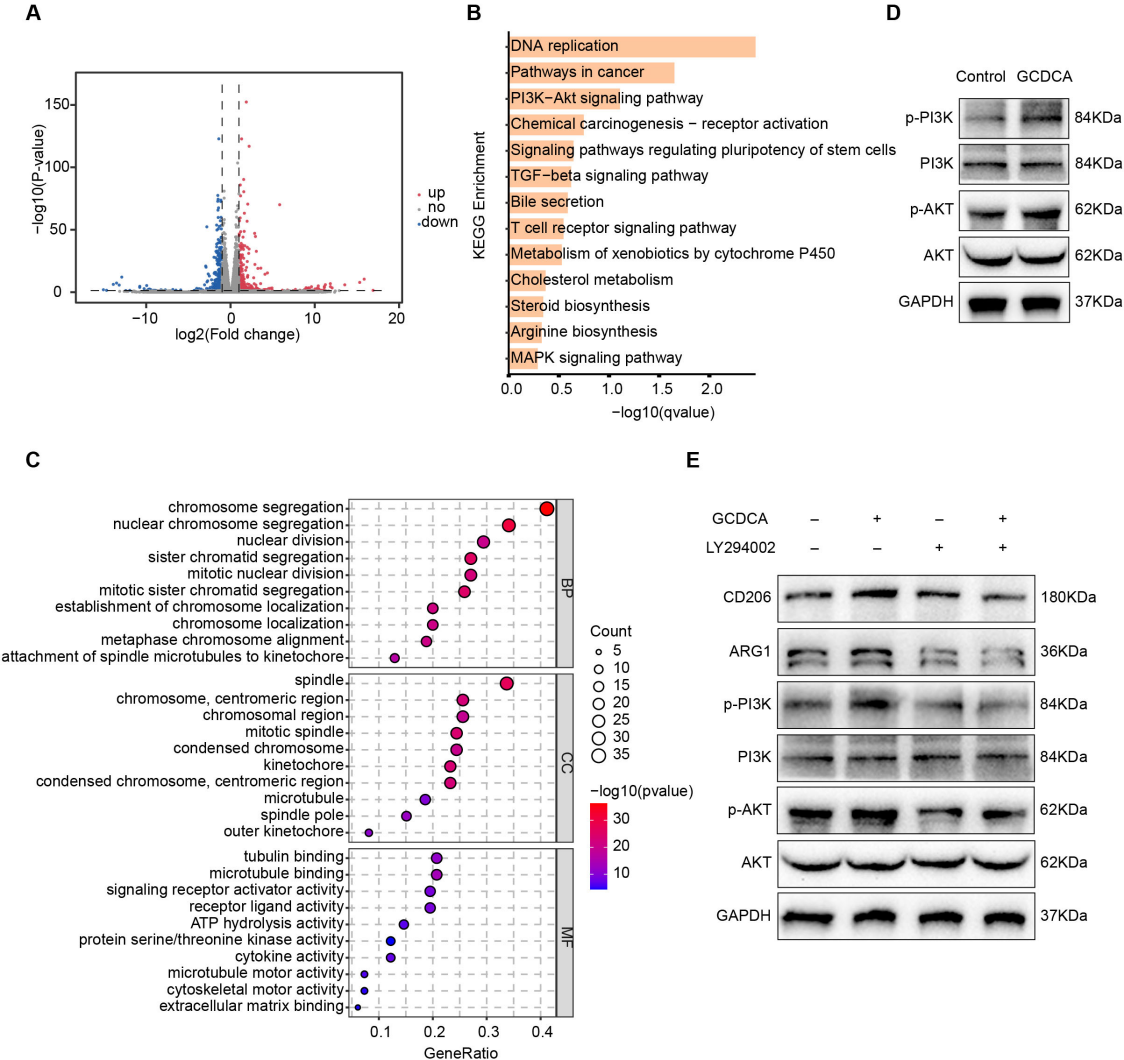
GCDCA induces macrophage polarization toward M2 via the S1PR2/PI3K/AKT signaling pathway. **(A)** Volcano plot of differentially expressed genes (DEGs) between GCDCA-treated and control groups (n = 3 in each group). **(B)** Histogram of KEGG enrichment results of DEGs between GCDCA-treated and control groups. **(C)** Bubble plots of GO enrichment results of DEGs between GCDCA-treated and control groups. **(D)** Western blotting for p-AKT, AKT, p-PI3K, and PI3K protein expression in GCDCA-treated NR8383 cells. **(E)** Western blotting was used to detect the expression of p-AKT, AKT, p-PI3K, PI3K, CD206, and ARG1 proteins after pretreatment with LY294002 inhibitor.

Previous studies have demonstrated that GCDCA induces M2-type macrophage polarization via S1PR2, which, consequently, enhances tumor cell stemness; however, the specific mechanism remains unclear. Although the number of studies on the mechanism of BA in the tumor immune microenvironment has increased in recent years, the specific mechanism by which GCDCA regulates M2-type macrophage polarization via S1PR2 receptors remains unclear. To explore the changes in gene expression in GCDCA-induced macrophages, we performed a transcriptome sequencing analysis of GCDCA-treated NR8383 cells and screened for genes with significant differences in expression. Volcano plots showed up- regulated and down-regulated differential genes after GCDCA treatment (**Figure 9A**). KEGG and GO enrichment analysis of all up-regulated differential genes revealed that the pathways enriched for up-regulated differential genes after GCDCA treatment were the PI3K/AKT signaling pathway and the TGF-β signaling pathway, among others (**Figures 9B, C**). Activation of the PI3K/AKT signaling pathway induces M2-type macrophage polarization (44). NR8383 cells were induced using GCDCA to investigate whether GCDCA functions through this pathway. The levels of AKT, p-AKT, PI3K, and p-PI3K proteins in the cells were detected by Western blotting, which showed significantly upregulated levels of p-AKT and p-PI3K proteins after GCDCA treatment (**Figure 9D**). Pretreatment of cells with the PI3K inhibitor LY294002 significantly downregulated p-AKT and p-PI3K protein levels as well as protein levels of the M2 macrophage markers CD206 and ARG1 (**Figure 9E**). These results indicate that GCDCA induces macrophage polarization toward the M2 type through the S1PR2/PI3K/AKT signaling pathway.

## 4 Discussion

Hepatocellular carcinoma (HCC) is a malignant tumor with high morbidity and mortality rates (45). Despite continuous improvements in therapeutic approaches in recent years, HCC treatment still faces great challenges owing to its complex immune microenvironment and the high heterogeneity of tumor cells. Recent studies have revealed the critical role of the TME in HCC progression, especially the influence of the polarization process of TAM on CSC properties. This study provides new therapeutic targets and ideas for HCC by exploring the role of GCDCA in HCC, especially the mechanism of enhanced M2-type macrophage polarization and tumor cell stemness mediated by the S1PR2 receptor.

GCDCA is a hydrophobic bile salt that accumulates in cholestatic liver disease (46). Bile salts contribute to the development and progression of HCC via their receptors. Bile acids are often thought to be pro-cancerous; however, the effect of BA on cancer depends on the signaling pathways activated by BA in different cancers, which can also be understood as differences in bile acid receptor expression (47). For example, CDCA attenuates inflammatory damage in hepatocytes by activating bile acid nuclear receptor FXR, which inhibits hepatic inflammation (48). However, CDCA in the uterus primarily activates the membrane receptor TGR5 to promote endometrial cancer cell proliferation (49). In addition, the effects of different bile acid concentrations on the same tissue varied. GCDCA at a concentration of 200 μM promoted the invasion and metastasis of HCC cells (30) and induced apoptosis in rat primary hepatocytes at 50 μM (50). In this study, we further verified the promotional role of GCDCA in HCC progression, especially under conditions of jaundice symptoms and cholestasis, which are common in patients with HCC. GCDCA accelerated the progression of HCC by affecting the hepatic immune microenvironment. We found that GCDCA not only aggravated liver injury but also significantly promoted HCC progression, which was intricately related to its role in promoting HCC stemness property. We used immunohistochemical and immunofluorescence analyses to demonstrate that the expression of tumor stemness markers (EPCAM, SOX9, and KRT19) was significantly increased in GCDCA-treated liver tissues, further confirming that GCDCA promotes the malignant progression of HCC by enhancing tumor cell stemness. Therefore, we next investigated how GCDCA enhances the stemness of HCC cells. Does it act directly or indirectly on liver cancer cells, thus promoting stemness? In our study, cloning and spheroidization experiments demonstrated that GCDCA had no direct effect on the stemness of human and rat HCC cells. Therefore, we speculated that GCDCA may act indirectly by affecting other components of the TME.

Macrophages in the TME regulate tumor immune escape and promote tumor invasion and metastasis. Macrophages are categorized into two subtypes based on their function and cytokine secretion: M1 and M2 (51). M1-type macrophages mediate anti-tumor immune responses, whereas M2-type macrophages suppress immune responses and promote tumor growth, metastasis, and immune escape (52). In this study, we used single-cell RNA sequencing to reveal the strong interplay between M2-type macrophages and CSC in the TME of HCC. *In vitro* experiments revealed that GCDCA further promoted tumor cell stemness and tumor progression by inducing the polarization of M2-type macrophages. In particular, GCDCA promotes the accumulation of immunosuppressive cytokines in the TME by activating M2-type macrophages, leading to the immune escape of tumor cells. This suggests that the role of M2-type macrophages in promoting HCC progression by GCDCA is not negligible, and it may be a new target for HCC immunotherapy. In addition, existing studies have demonstrated that GCDCA can inhibit tumor-specific T cell responses in the microenvironment, reduce the immunotherapy effect of anti-programmed cell death protein 1 (anti-PD-1), and promote tumor progression (4). Simultaneously, GCDCA can activate autophagy in liver cancer cells, promoting the invasion and metastasis of liver cancer (30). An increase in GCDCA content in the intestinal microbiota causes lipid metabolism disorders and inflammatory responses, promoting the progression of metabolic dysfunction-associated fatty liver disease (MAFLD) (53). Therefore, GCDCA may exert extensive effects on different components of liver cancer cells and the TME, such as macrophages and T cells, and may jointly influence the occurrence and development of HCC through metabolic or non-immune-related pathways.

The S1PR2 receptor, a bile acid receptor, has been reported to mediate the progression of HCC in a mouse model of non-alcoholic steatohepatitis (54). Under cholestatic conditions, TCA mediates HSC activation via the S1PR2/p38 MAPK/YAP pathway (55). However, the role of S1PR2 in macrophage polarization has not been fully investigated. We demonstrated for the first time the critical role of S1PR2 in GCDCA-induced polarization of M2-type macrophages and enhanced stemness of tumor cells. Single-cell sequencing revealed that S1PR2 is widely expressed in M2-type macrophages. *S1PR2* positively correlated with M2 macrophage marker genes (*CD68*, *MRC1*, *CD163*) and stemness marker genes (*EPCAM*, *KRT19*, *SOX9*) in human HCC tissues. Further experimental results demonstrated that the inhibition of the S1PR2 receptor significantly reversed the GCDCA-induced polarization of M2-type macrophages and enhanced tumor cell stemness. This finding provides a new mechanism for GCDCA-mediated tumor progression and suggests that S1PR2 plays an important role in regulating tumor immune responses.

Emerging clinical evidence further confirms that TME heterogeneity is a key determinant of treatment response. Pan-cancer analysis revealed that specific molecules, such as nucleolar and spindle-associated protein 1 (PLIN3), killer cell lectin-like receptor B1 (KLRB1), and ephrin receptor B2 (EPHB2), are not only associated with poor prognosis but also serve as potent markers for assessing M2 macrophage infiltration. Their expression is correlated with reduced survival rates in multiple cancers, including lung adenocarcinoma and breast cancer (56–58). Additionally, disulfidptosis-related genes can stratify the tumor immune microenvironment and predict immunotherapy responses in melanoma, lung adenocarcinoma, and triple-negative breast cancer. A recent study aimed to reshape targeted therapeutic strategies for the TME. For instance, the αCD73-PLG-MMAE conjugate for triple-negative breast cancer can simultaneously induce immunogenic cell death and disrupt immunosuppressive adenosine signaling, thereby restoring durable antitumor immune responses. Collectively, these studies revealed dynamic interactions among immune cells, tumor cells, and the broader TME, providing a robust framework for predicting outcomes and developing novel combination immunotherapies against cancer (59, 60).

The role of GCDCA in the genesis and development of HCC and in the immune microenvironment has gradually become a popular research topic. Although further clinical studies are warranted to confirm the specific mechanism and therapeutic potential, the relationship between GCDCA and HCC provides new ideas and possibilities for the diagnosis and treatment of liver diseases. Future studies should focus on elucidating the direct effects of GCDCA on liver macrophages and how it contributes to the development and progression of HCC through signaling pathways, immune regulation, and metabolic alterations.

## Data Availability

The single-cell RNA sequencing data generated in this study are available at the Gene Expression Omnibus (GEO, https://www.ncbi.nlm.nih.gov/geo/query/acc.cgi?acc=GSE218561), RNAsequencing data are available from the corresponding author on reasonable request. Other relevant data are within the manuscript and its additional files.

## Supplementary material

Supplementary Figure 1 (A) Quantitative analysis of representative immunohistochemical staining of KRT19, EPCAM and SOX9 positive cells in the livers of different groups of rats in **Figure 2I**. ****p* < 0.001

Supplementary Figure 2 (A) Representative histograms and quantitative mean fluorescence intensity (MFI) of CD206 expression on THP-1 derived macrophages under different conditions: M0 (un-polarized state), M2 (polarized with IL-4/IL-13), and the M2 group treated with GCDCA. (B) Representative histograms and quantitative mean fluorescence intensity (MFI) of CD163 expression on THP-1 derived macrophages under different conditions: M0 (un-polarized state), M2 (polarized with IL-4/IL-13), and the M2 group treated with GCDCA. (C) ELISA analysis of TNF-α, IL-6, IL-10, TGF-β, and VEGF in supernatants from NR8383 (Control, Control+GCDCA). (D) ELISA analysis of TNF-α, IL-6, IL-10, TGF-β, and VEGF in supernatants from THP-1-derived macrophages under indicated conditions (M0, M2, M2+GCDCA). **p* < 0.05, ***p* < 0.01, and ****p* < 0.001, n.s. = not significant.

Supplementary Figure 3 (A) Quantitative analysis of representative immunohistochemical staining of CD68, CD163 and S1PR2 positive cells in the livers of different groups of rats in **Figure 6E**. ****p* < 0.001

Supplementary Figure 4 (A) Quantitative analysis of representative immunohistochemical staining of KRT19, EPCAM and SOX9 positive cells in the livers of different groups of rats in **Figure 7I**. (B) Effects of JTE-013 (10 μmol/L) treatment at different time points on the relative cell viability of hepatocellular carcinoma cells (Huh7/HepG2) (*n* = 3 in each group). (C) Effect of JTE-013 (10 μmol/L) on clonogenic ability of different hepatocellular carcinoma cell lines (Huh7/HepG2). ***p* < 0.01, and ****p* < 0.001, n.s. = not significant.

Supplementary Figure 5 (A) Expression of M2 macrophage marker genes Mrc1, Cd163, and Arg1 in NR8383 cells after inhibition of PXR receptor (*n* = 3 in each group). (B) Expression of CD206, CD163, and ARG1 proteins in NR8383 cells after inhibition of the PXR receptor. (C) Expression of M2 macrophage marker genes MRC1, ARG1, and TGF-β in THP-1 cells after inhibition of PXR receptor (*n* = 3 in each group). (D) Expression of CD206, CD163, and ARG1 proteins in THP-1 cells after inhibition of the PXR receptor. (E) Expression of M2 macrophage marker genes Mrc1, Cd163, and Arg1 in NR8383 cells after inhibition of VDR receptor (*n* = 3 in each group). (F) Expression of CD206, CD163, and ARG1 proteins in NR8383 cells after inhibition of the VDR receptor. (G) Expression of M2 macrophage marker genes MRC1, ARG1, and TGF-β in THP-1 cells after inhibition of VDR receptor (*n* = 3 in each group). (H) Expression of CD206, CD163, and ARG1 proteins in THP-1 cells after inhibition of the VDR receptor. ***p* < 0.01, and ****p* < 0.001, n.s. = not significant.
