# Supplementary figures and images for: GCDCA promotes hepatocellular carcinoma progression through S1PR2/PI3K/AKT-mediated polarization of M2-type macrophages
Source: Front Immunol. 2026 Feb 23;17:1640450. doi: 10.3389/fimmu.2026.1640450 (PMC12968304; doi:10.3389/fimmu.2026.1640450)

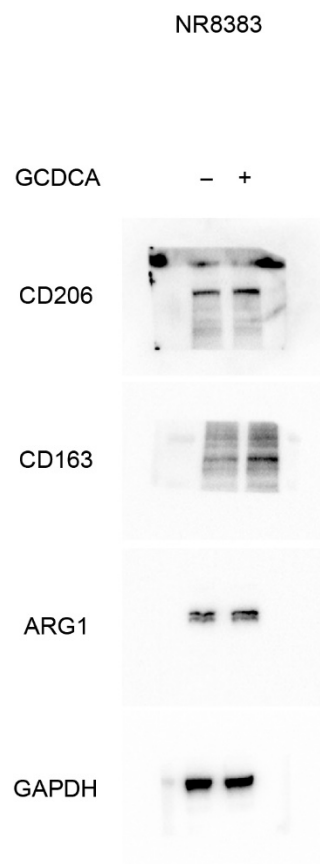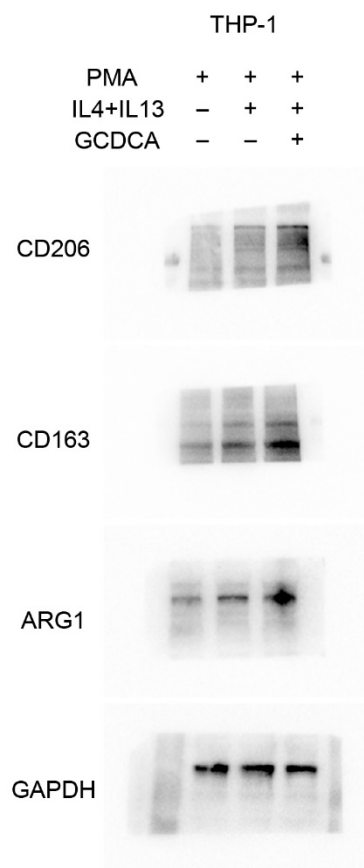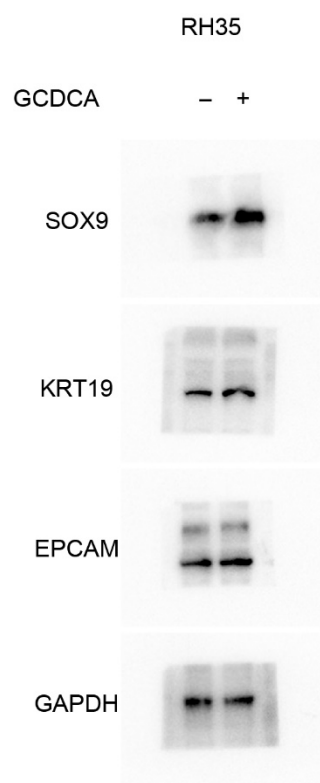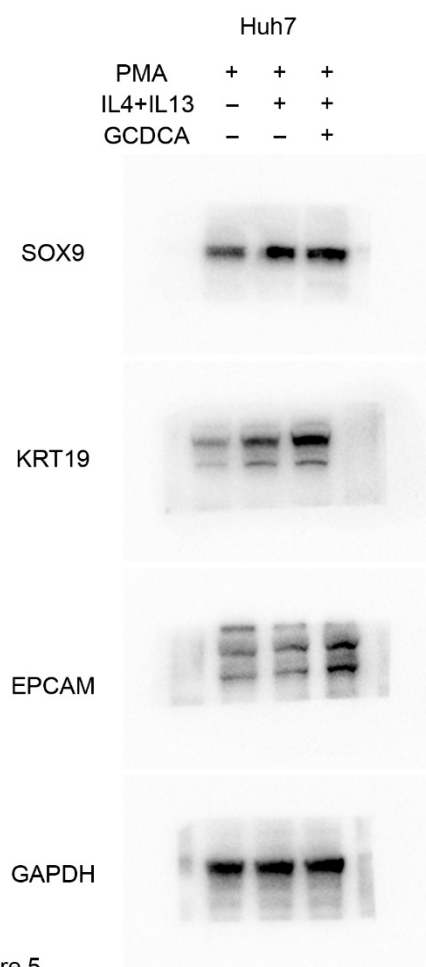

Refer to Figure 5

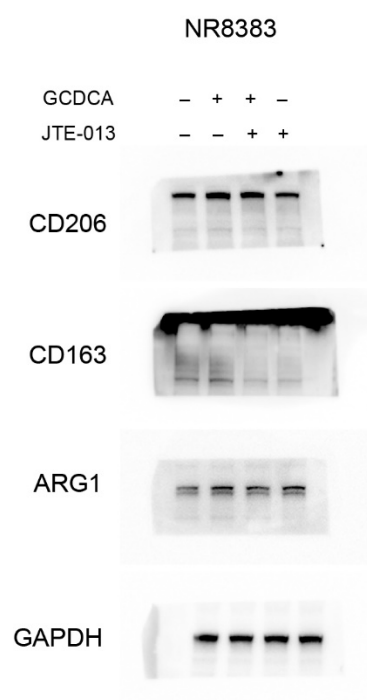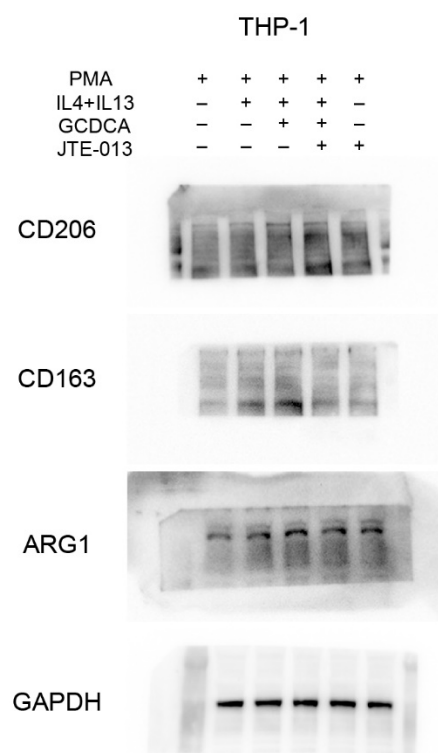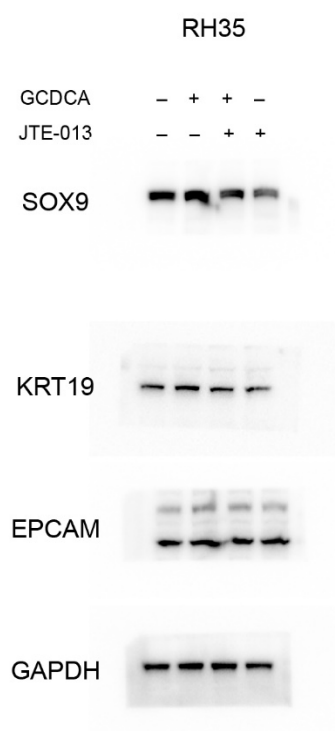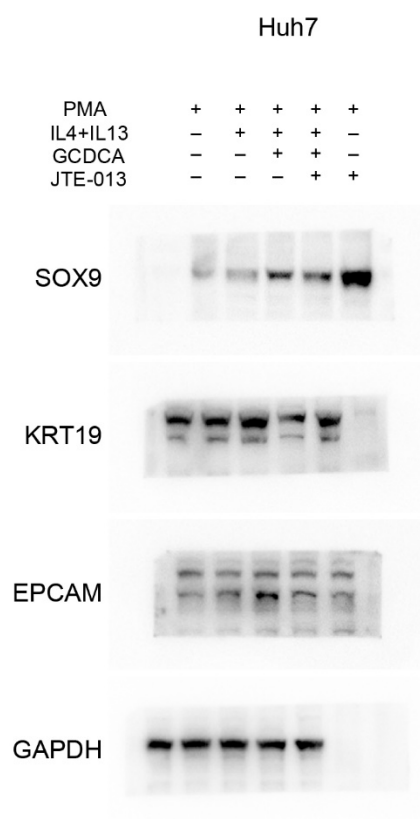

Refer to Figure 8

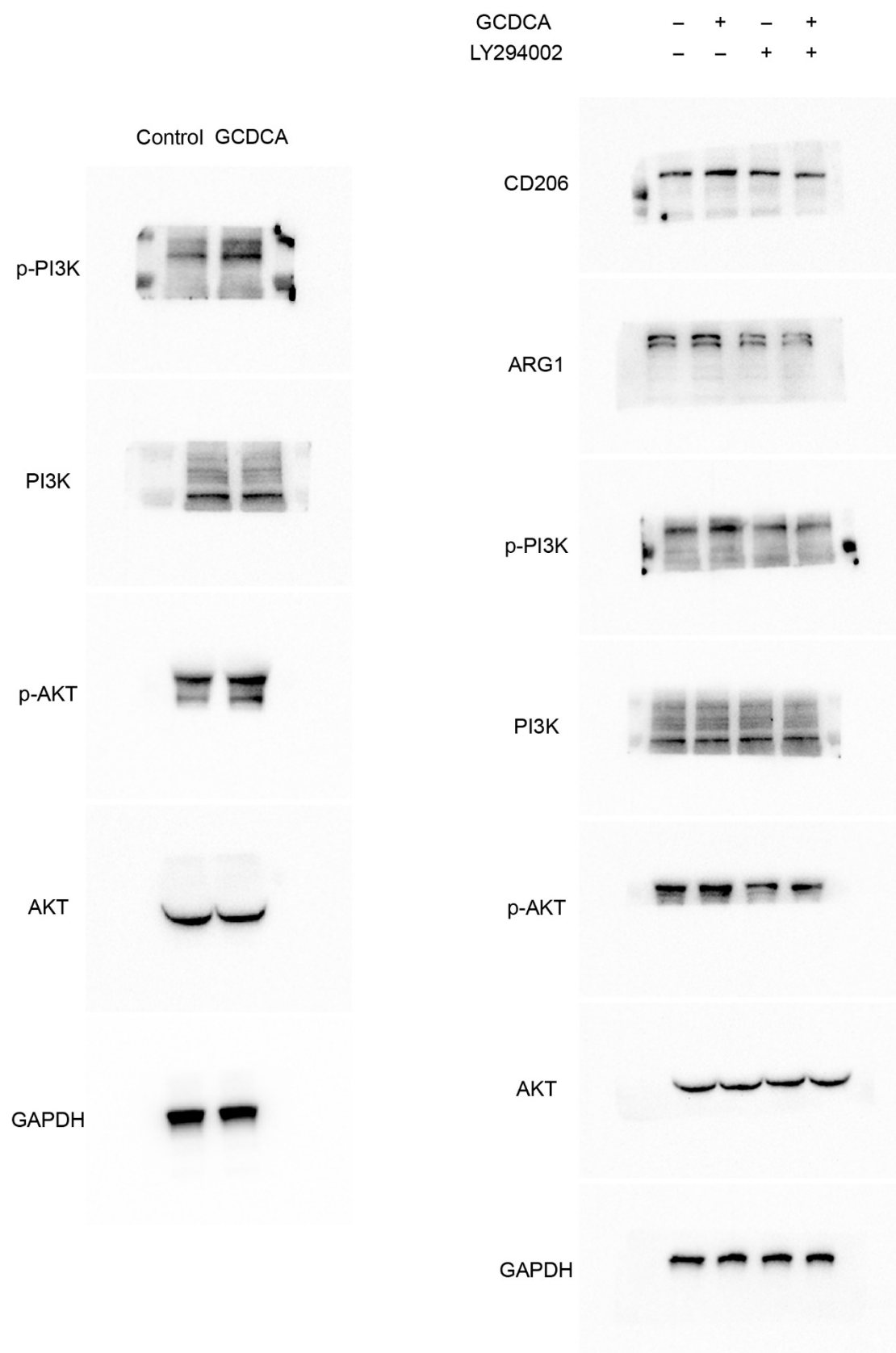

Refer to Figure 9

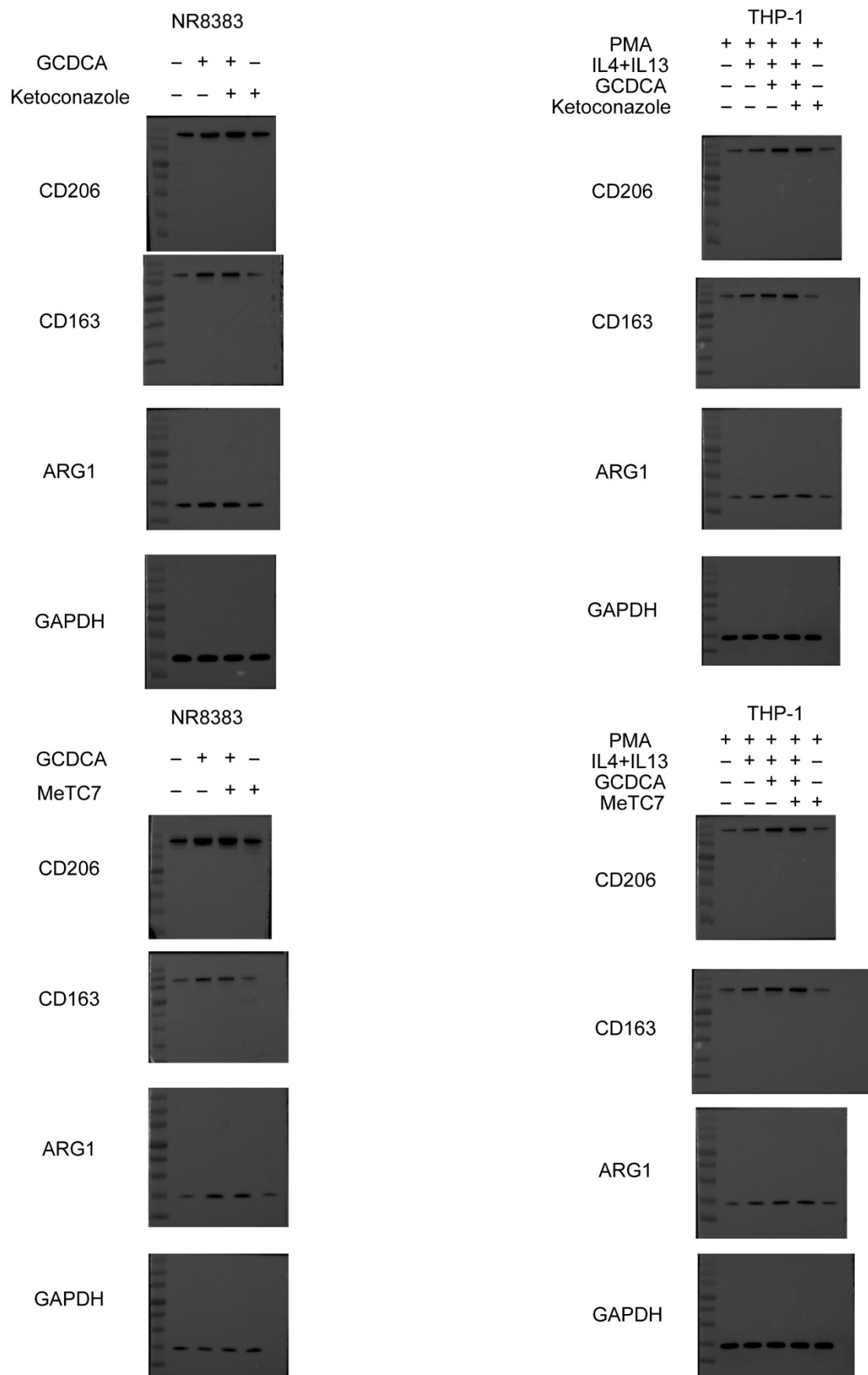

Supplement: Supplementary file 1 [file DataSheet1.pdf]

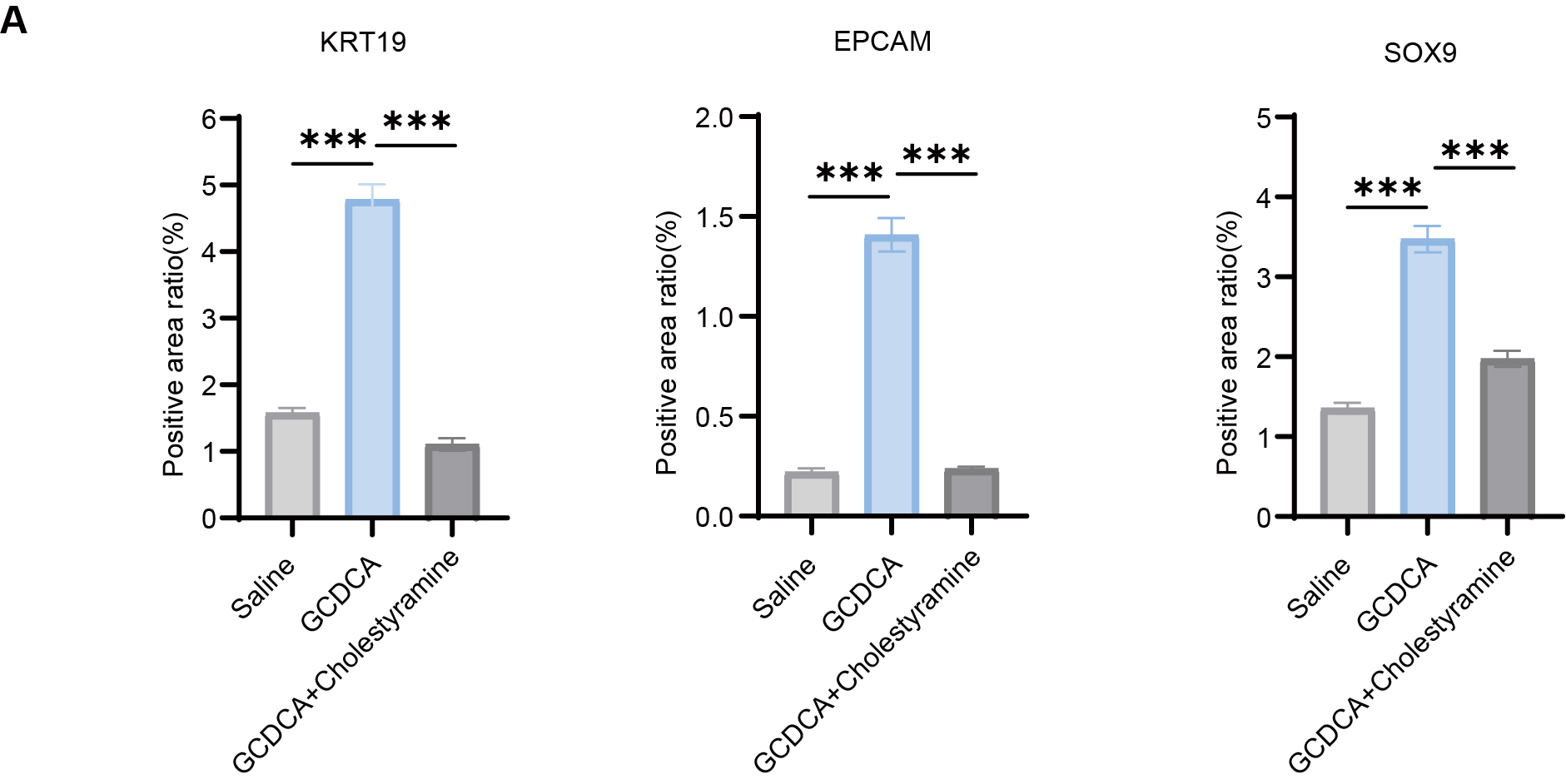

Supplement: Supplementary Figure 1 — (A) Quantitative analysis of representative immunohistochemical staining of KRT19, EPCAM and SOX9 positive cells in the livers of different groups of rats in Figure 2I. ***p < 0.001 [file Image1.jpeg]

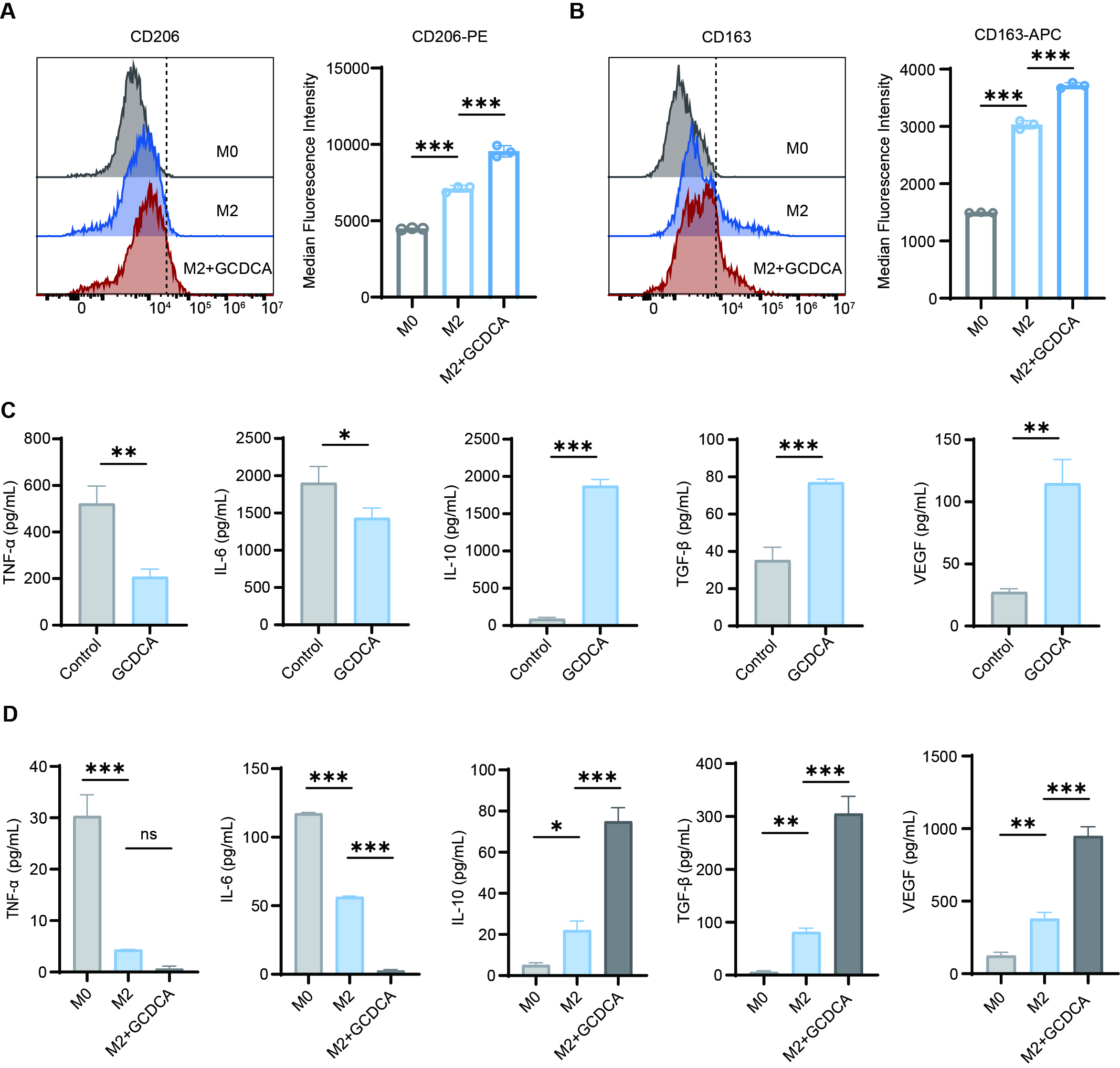

Supplement: Supplementary Figure 2 — (A) Representative histograms and quantitative mean fluorescence intensity (MFI) of CD206 expression on THP-1 derived macrophages under different conditions: M0 (un-polarized state), M2 (polarized with IL-4/IL-13), and the M2 group treated with GCDCA. (B) Representative histograms and quantitative mean fluorescence intensity (MFI) of CD163 expression on THP-1 derived macrophages under different conditions: M0 (un-polarized state), M2 (polarized with IL-4/IL-13), and the M2 group treated with GCDCA. (C) ELISA analysis of TNF-α, IL-6, IL-10, TGF-β, and VEGF in supernatants from NR8383 (Control, Control+GCDCA). (D) ELISA analysis of TNF-α, IL-6, IL-10, TGF-β, and VEGF in supernatants from THP-1-derived macrophages under indicated conditions (M0, M2, M2+GCDCA). *p < 0.05, **p < 0.01, and ***p < 0.001, n.s. = not significant. [file Image2.jpeg]

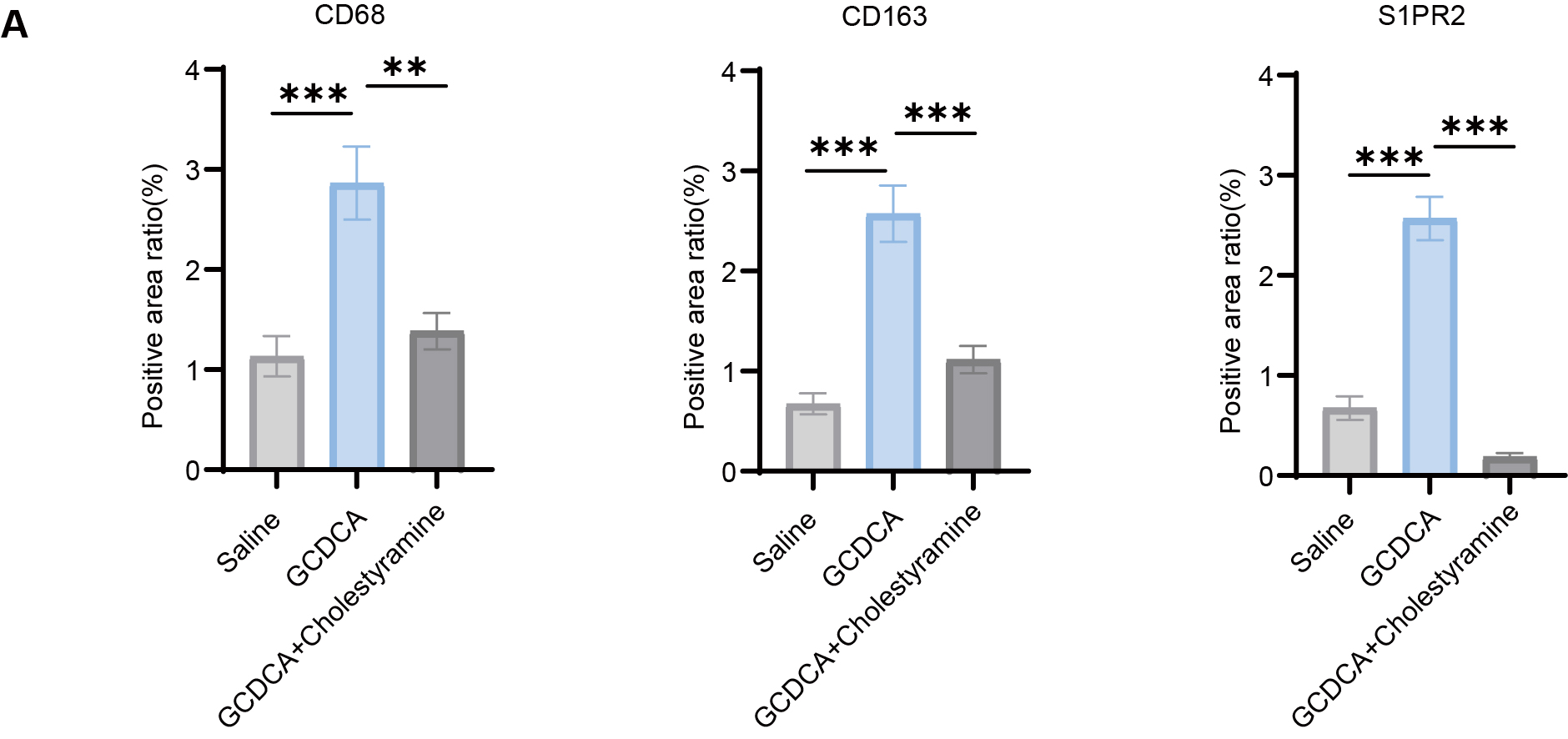

Supplement: Supplementary Figure 3 — (A) Quantitative analysis of representative immunohistochemical staining of CD68, CD163 and S1PR2 positive cells in the livers of different groups of rats in Figure 6E. ***p < 0.001 [file Image3.jpeg]

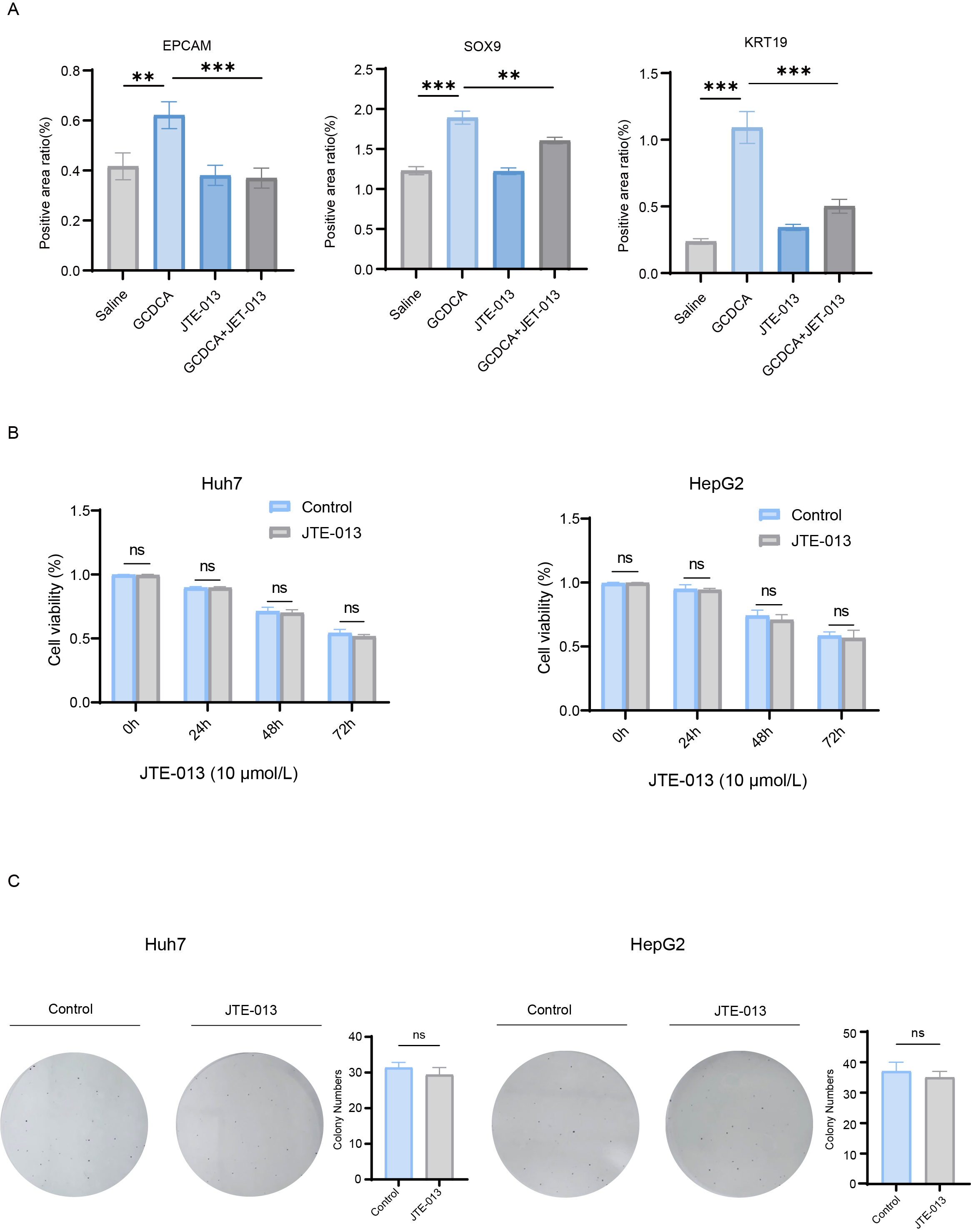

Supplement: Supplementary Figure 4 — (A) Quantitative analysis of representative immunohistochemical staining of KRT19, EPCAM and SOX9 positive cells in the livers of different groups of rats in Figure 7I. (B) Effects of JTE-013 (10 μmol/L) treatment at different time points on the relative cell viability of hepatocellular carcinoma cells (Huh7/HepG2) (n = 3 in each group). (C) Effect of JTE-013 (10 μmol/L) on clonogenic ability of different hepatocellular carcinoma cell lines (Huh7/HepG2). **p < 0.01, and ***p < 0.001, n.s. = not significant. [file Image4.jpeg]

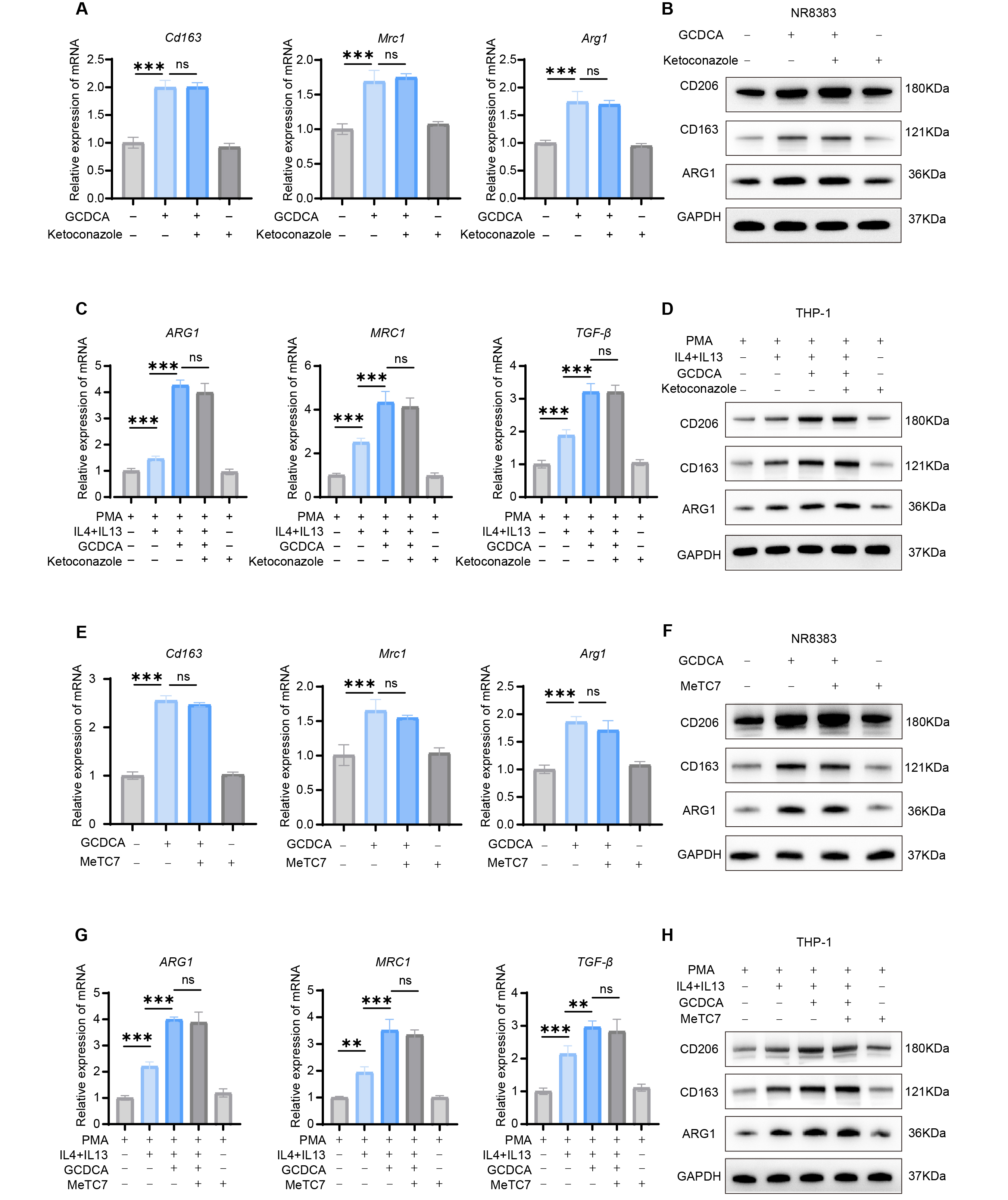

Supplement: Supplementary Figure 5 — (A) Expression of M2 macrophage marker genes Mrc1, Cd163, and Arg1 in NR8383 cells after inhibition of PXR receptor (n = 3 in each group). (B) Expression of CD206, CD163, and ARG1 proteins in NR8383 cells after inhibition of the PXR receptor. (C) Expression of M2 macrophage marker genes MRC1, ARG1, and TGF-β in THP-1 cells after inhibition of PXR receptor (n = 3 in each group). (D) Expression of CD206, CD163, and ARG1 proteins in THP-1 cells after inhibition of the PXR receptor. (E) Expression of M2 macrophage marker genes Mrc1, Cd163, and Arg1 in NR8383 cells after inhibition of VDR receptor (n = 3 in each group). (F) Expression of CD206, CD163, and ARG1 proteins in NR8383 cells after inhibition of the VDR receptor. (G) Expression of M2 macrophage marker genes MRC1, ARG1, and TGF-β in THP-1 cells after inhibition of VDR receptor (n = 3 in each group). (H) Expression of CD206, CD163, and ARG1 proteins in THP-1 cells after inhibition of the VDR receptor. **p < 0.01, and ***p < 0.001, n.s. = not significant. [file Image5.jpeg]
