# Supplementary material for: GCDCA promotes hepatocellular carcinoma progression through S1PR2/PI3K/AKT-mediated polarization of M2-type macrophages
Source: Front Immunol. 2026 Feb 23;17:1640450. doi: 10.3389/fimmu.2026.1640450 (PMC12968304; doi:10.3389/fimmu.2026.1640450)
Supplement: Supplementary file 2 [file Table1.docx]

Supplementary table 1. Primers used in this study.

| 引物名称 |  | 引物序列（5’-3’） | |
| --- | --- | --- | --- |
| *h-EPCAM* | Forward Primer | | TTGTGGTTGTGGTGATAGCAGTTG |
|  | Reverse Primer | | ACCCATCTCCTTTATCTCAGCCTTC |
| *h-KRT19* | Forward Primer | | AAGATCCTGAGTGACATGCGAAGC |
|  | Reverse Primer | | AGTAACCTCGGACCTGCTCATCTG |
| *h-SOX9* | Forward Primer | | GGCAGGCGGAGGCAGAGG |
|  | Reverse Primer | | GGAGGAGGAGTGTGGCGAGTC |
| *h-MRC1* | Forward Primer | | TTCAGTGGACCATCGAGGAAGAGG |
|  | Reverse Primer | | ATGGCAACACACCCTGGCTTTC |
| *h-TGFβ* | Forward Primer | | CTAATGGTGGAAACCCACAACG |
|  | Reverse Primer | | TATCGCCAGGAATTGTTGCTG |
| *h-ARG1* | Forward Primer | | GTTGACGGACTGGACCCATCTTTC |
|  | Reverse Primer | | GCAACTGCTGTGTTCACTGTTCG |
| *h-GAPDH* | Forward Primer | | AGTCCACTGGCGTCTTCACC |
|  | Reverse Primer | | TGATCTTGAGGCTGTTGTCATACTTC |
| *r-Epcam* | Forward Primer | | CTTGGTGATGAAGGCGGAGATGAC |
|  | Reverse Primer | | CGTTGCACTGCTTGGCTTTGAAG |
| *r-Krt19* | Forward Primer | | AGGCACTGTGGCAGAGATAGAGG |
|  | Reverse Primer | | TGTGTCAGCACGCACGTTACTC |
| *r-Sox9* | Forward Primer | | TCAACGGCTCCAGCAAGAACAAG |
|  | Reverse Primer | | CCGCTCCGCCTCCTCCAC |
| *r-Mrc1* | Forward Primer | | TGGACAGACGGACGAGGAGTTC |
|  | Reverse Primer | | GCCACCAATCACAACAACACAGTC |
| *r-Cd163* | Forward Primer | | TCACAGCATGGCACAGGTCATTC |
|  | Reverse Primer | | GTCGTCGCTTCAGAGTCCACAAG |
| *r-Arg1* | Forward Primer | | AGTGTGGTGCTGGGTGGAGAC |
|  | Reverse Primer | | GCGGAGTGTTGATGTCAGTGTGAG |
| *r-Gapdh* | Forward Primer | | CAACTCCCTCAAGATTGTCAGCAA |
|  | Reverse Primer | | GGCATGGACTGTGGTCATGA |
